# Application of the Finite Element Method in the Analysis of Composite Materials: A Review

**DOI:** 10.3390/polym12040818

**Published:** 2020-04-04

**Authors:** Sarah David Müzel, Eduardo Pires Bonhin, Nara Miranda Guimarães, Erick Siqueira Guidi

**Affiliations:** 1 Department of Materials and Technology, School of Engineering, São Paulo State University (Unesp), 12.516-410 Guaratinguetá, São Paulo, Brazil; eduardo.bonhin@unesp.br (E.P.B.); nara.guimaraes@unesp.br (N.M.G.); 2 Department of Mechanical, School of Engineering, São Paulo State University (Unesp), 12.516-410 Guaratinguetá, São Paulo, Brazil; erick.s.guidi@unesp.br

**Keywords:** anisotropic material, orthotropic material, transverse isotropic material, multiscale approaches, failure criteria, plate element, shell element

## Abstract

The use of composite materials in several sectors, such as aeronautics and automotive, has been gaining distinction in recent years. However, due to their high costs, as well as unique characteristics, consequences of their heterogeneity, they present challenging gaps to be studied. As a result, the finite element method has been used as a way to analyze composite materials subjected to the most distinctive situations. Therefore, this work aims to approach the modeling of composite materials, focusing on material properties, failure criteria, types of elements and main application sectors. From the modeling point of view, different levels of modeling—micro, meso and macro, are presented. Regarding properties, different mechanical characteristics, theories and constitutive relationships involved to model these materials are presented. The text also discusses the types of elements most commonly used to simulate composites, which are solids, peel, plate and cohesive, as well as the various failure criteria developed and used for the simulation of these materials. In addition, the present article lists the main industrial sectors in which composite material simulation is used, and their gains from it, including aeronautics, aerospace, automotive, naval, energy, civil, sports, manufacturing and even electronics.

## 1. Introduction

Technological advancements have led to an increase in the demand of special materials with unique properties that cannot be found in metal alloys, ceramics or polymers blends [1,2].

To supply these needs, composite materials were developed. They are made of two or more distinctive and immiscible materials with different mechanical, physical and/or chemical properties [1,3,4,5].

Composites are considered heterogeneous and multiphase engineered materials, in which the matrix is responsible for binding the reinforcement together and transferring the loads between the fibers, while the reinforcement adds rigidity and obstructs crack propagation in the structure [1,6,7,8,9].

They can be classified according to the matrix (metallic, polymeric and ceramic) or the type of reinforcement used (fibers or particles) [1,7,8,10]. The ones with a polymeric matrix and continuous fibers have great relevance and significance, due to their excellent mechanical properties, good thermal stability and low density [11].

**Figure 1:**
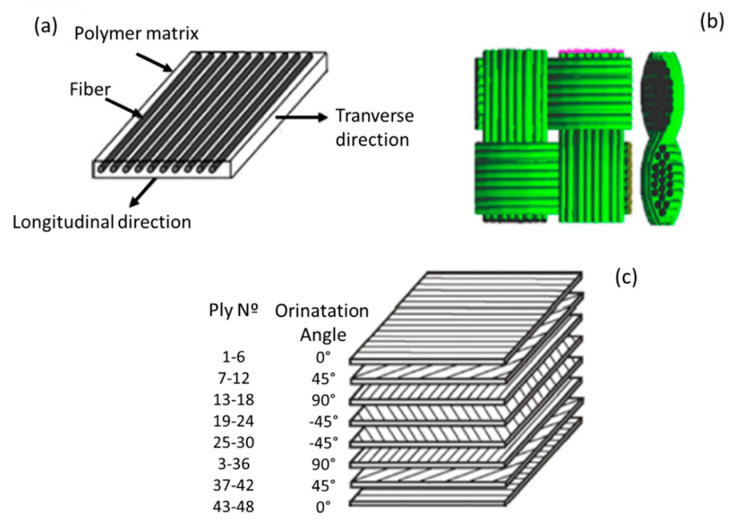
Scheme of the most common uses of fiber reinforced composite structures: (**a**) unidirectional fiber orientation ply, (**b**) bidirectional fiber orientation ply (woven-ply) and (**c**) multiorientation laminate, quasi-isotropic laying-up sequence [0°/45°/90°/−45°] 6S. Reproduced with permission [13].

Fiber reinforcements can be either unidirectional (UD) (Figure 1a) or bidirectional (Figure 1b). Unidirectional fibers have maximum stiffness and strength along the fiber direction and minimal properties in the transverse direction, exhibiting anisotropy. Bidirectional reinforcements have maximum stiffness and strength in the fiber direction [12,13]. Unidirectional fibers can be aligned on a thin plate; pre-impregnated with resin; and used to define the stacking order and layer design for composite laminates (Figure 1c). The bidirectional reinforcements are woven fabrics, and there are several types of weaving [12,13].

Anisotropic materials show different mechanical properties in each direction; i.e., they are not symmetrical with respect to all their planes or axes. Orthotropic materials are a subset of anisotropic materials that show a symmetry between two planes, in general, the plane parallel to the fibers has significantly superior properties compared to the orthotropic perpendicular plane. Wood is a good example of an orthotropic material; its properties perpendicular to the fiber axis (radial and tangential) are worse than its parallel ones. In this case, the properties of radial and tangential directions are not equal, but similar, being equally inferior to the longitudinal direction.

Composites have a set of performance characteristics that their constituents cannot achieve by themselves individually [5,7]. Due to these combinations, it is possible to obtain lightweight design with high strength and stiffness; some other key characteristics are high-temperature, corrosion and impact resistance. Together, said things make composites more interesting, useful and attractive alternatives [14,15,16,17].

Because of these characteristics, composites are widely applied in automotive, aeronautical, petrochemical, naval, electro-electronic, civil construction, energy, biomedical, sports and manufacturing industries, among others [1,5,14,15,18,19,20]. Their applications can be seen in several industrial sectors; however, they are expensive and difficult to characterize due to their heterogeneity and laminate configurations, which affect their final properties.

Owing to this difficulty and their toughness, to optimize and improve structural projects, as well as to understand better the behaviors of these materials, some researchers resort to computational simulations. Through the use of the finite element method (FEM), it is possible to understand the damages caused in the matrix, the fiber and their interface when the composite is exposed to severe conditions, such as static and dynamic loading, different temperatures and pressures, among others [21,22].

Analytical models are not always able to sufficiently address all failure phenomena that contribute to composite performance [23]. Different failure mechanisms play important roles during the service-lives of composite materials; for example, fracturing of the reinforcement is a partial detachment of the interface, which results in the nucleation and growth of voids, and their coalescence in the matrix.

According to Lasri et al. [24], damage mechanisms in composite materials generally include four types of failure modes: transverse matrix fracture, fiber–matrix interface detachment, fiber rupture and layer delamination. In general, transverse fracture of the matrix is the first damage process to occur, since the matrix has lower failure stress compared to all laminate constituents. Fiber–matrix interface detachment can accompany transverse fracture of the matrix and facilitate its progression [24,25]. Transverse failure can happen without breaking any longitudinal fiber. Such failures are parallel to the fibers and lead to a decrease in stiffness. Thus, damage criteria are required to indicate the onset of failure and damage orientation.

Note that when applying FEM to composites, it is important to consider some particularities of these materials, such as the constitutive law; modelling and failure criteria associated with the composites; and the type of elements used to model the objects.

## 2. Modelling

For the simulation of composites, three primary approaches are usually applied, those being: (a) a micromechanics-based approach, (b) an equivalent homogeneous material (EHM) based approach and (c) a combination of the two previous approaches. Notice that each method has advantages and disadvantages [23,26,27]. 

According to Dandekar and Shin [23] the micromechanical based approach describes the material behavior locally, and thereby, it is possible to study local defects, such as fiber–matrix detachment and complex deformation mechanisms, especially in fiber reinforced composites. However, the time required to solve a simulation is very high, because the mesh used is very fine compared to the EHM model.

The EHM approach reduces simulation time, but it is not able to predict local effects; e.g., damage at the fiber–matrix interface [23,28,29]. Dandekar and Shin [23] said that it is possible to take advantage of the two models, and their ability to predict shear force and sub-surface damage.

**Figure 2:**
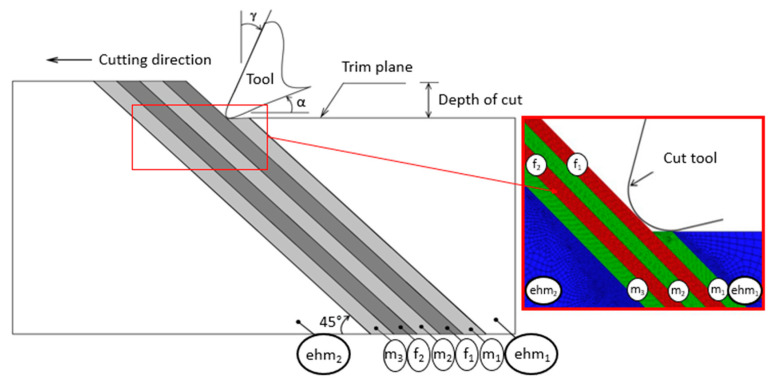
Schematic view of the fiber, matrix and equivalent homogeneous material (EHM) domains used in a finite element model for the case of 45° fiber orientation. Reproduced with permission [30].

Venu Gopala Rao, Mahajan and Bhatnagar [30] applied this methodology in their work. They studied the machining of the UD-GFRP composite’s chip formation mechanism. For that, the portion of the work material adjacent to the cutting tool was modelled—fiber and matrix separately—whereas portions away from the cutting tool were modelled as EHM (Figure 2).

For them, the most important thing was to study the cutting zone and that is why they modelled this region with the specification of a composite. As for the tool and the region around the cutting zone, single results were not so important to them, those being analyzed with the EHM model.

Jones [31] noted that these approaches define how rigidity and strength properties are chosen for the project materials. A composite material’s behavior can be separated into the micro and the macroscale, defined as follows:

➢ Microscale—study the composite material’s behavior, for which interactions from constituent materials are examined in detail as defined by heterogeneous material behavior.
➢ Macroscale—study the composite material behavior considered to be homogeneous, and the effects of all constituent materials are detected only by the composite material’s mean apparent properties.

Tenek and Argyris [22] went further in their conjectures; they cited that these questions address two fundamental problems: how to define the sheet properties using microscale procedures, and how to apply these properties on a macroscale for a global analysis.

At the microscale there are many difficulties due to the uncertainties that may require stochastic or statistical models. The objective of most approaches is to define the composite modules from all constituent materials or the strengths of the composite in terms of its phases. Therefore, some basic approaches include using the materials’ mechanisms and elasticity theories based on the repetition of a unit cell or some other representative volume, assuming that there is a perfect bond between fibers and matrix, which may not be true most of the time. Frequently, micro-mechanical theories are validated with experimental work [22,31,32].

**Figure 3:**
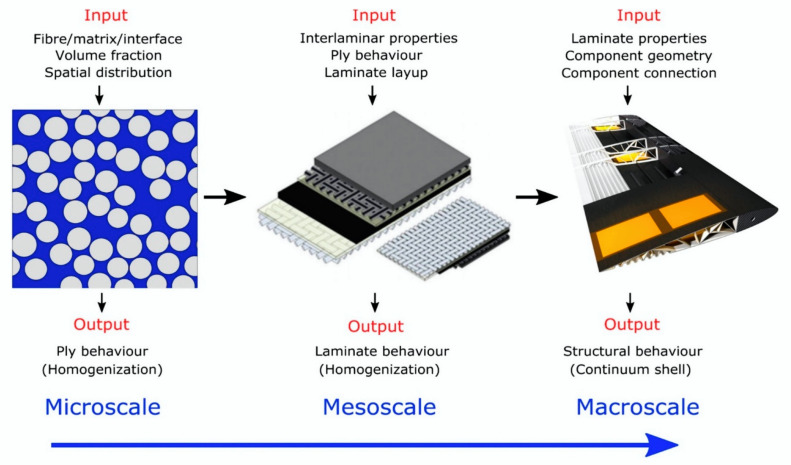
From the microscale to the macroscale. Reproduced with permission [33].

In Figure 3 is shown a structural evolution scheme for the hierarchy of a unit cell starting from a ply to a global composite structure.

In the simulation study of composites, it is possible to evaluate properties from nanoscale to macroscale, or in other words, to apply the multiscale technique, which consists of simulating the behavior of a composite through multiple time and/or length scales [34,35,36]. Some applications of these techniques are mainly focused on microstructural and mechanical property simulations of many classes of materials, including nanocomposites [35,37,38,39,40].

**Figure 4:**
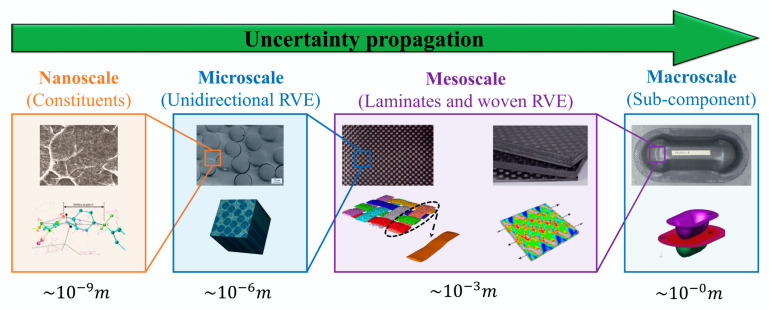
Schematic view of a four-scale woven fiber composite with polymer matrix: In computational modeling of this structure, each integration point at any scale is a realization of a structure at a finer scale. Due to the delicacy of materials at fine-scales, RVEs at lower scales may embody more uncertainty than those at higher scales. To quantify the uncertainty in a macroscopic quantity of interest, the relevant uncertainty sources at the lower scales should be identified for uncertainty propagation. Reproduced with permission [41].

Usually, both micro and macroscale approaches are extensively applied, but with the increase in nanomaterials as polymer composite reinforcements, nanoscale has been gaining ground. Coupled with the need for a better understanding of damage progression, mesoscale is thus an optimal option (Figure 4).

Multiscale techniques improve classical method solutions and analysis; i.e., solving local problems and taking this information from the smallest scale to the highest level, in a hierarchical process, through homogenization technique [42]. Aboudi, Arnold and Bednarcyk [34] explain that the homogenization technique provides the properties or responses of a "structure" (higher level) when given the properties or responses of the structure "constituents" (smaller scale). In summary, information from lower scale levels is used by higher scale levels to investigate the effects of constituents and microstructure on the mechanical properties, and those of the structural performances of the parts on macroscale properties [43,44]. 

Shaik and Salvi [45] commented that in studies of large structures, which have millions of components; materials and nonlinear structures; and many joints, it is impossible to model all of these details due to computational limitations. In such a case, a multiscale modelling approach is used when only a particular area of interest needs to be modeled with precision and in detail, saving a lot of time and computing power. However, the authors emphasized that these methodologies require experience and careful judgment by the designer.

In this approach, a geometry, with a repeating unit cell (RUC) and all of it constituents, is developed to build a representative volume element (RVE), along with its components and their interactions, to be modeled. The structure is shaped based on RUC/RVE and analyzed on different length scales with the desired confidence level, adding damage and failure aspects. The results undergo qualitative and quantitative evaluations from the material, configurational and architectural perspectives [34,45].

Shaik and Salvi [45] explained that the project can be divided into several scale sizes, depending on the confidence of the associated scale theory and the level of interest; i.e., the scale could be divided into micro (RUC), meso (RVE) and macroscale. At the micro level, the study focuses on the fiber: composition, geometry and orientation within the RUC. At meso level, mechanical characteristics of the material built from many of RUCs are studied, which produce homogeneous properties independent of any final effect and influence from the properties of the components. Finally, macro level includes mesoscale properties when the laws of continuous mechanics are applied. By connecting these scales, the performances of macroscale structures can be related to microscale individual constituents, such as the fiber, the resin and their interface.

**Figure 5:**
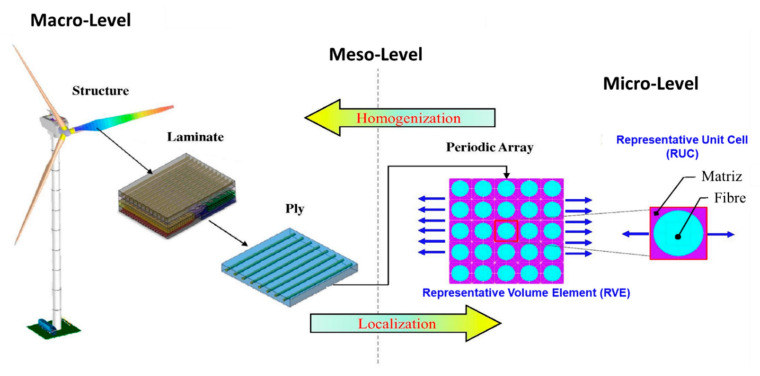
Schematic of multiscale modelling of engineering composite structures. Reproduced with permission [46].

Figure 5 presents a schematic of multiscale modeling used to analyze the blade of a wind turbine, showing the use of the microscale, mesoscale and macroscale, and the example of the application of RCU and RVE of a unidirectional laminate.

**Figure 6:**
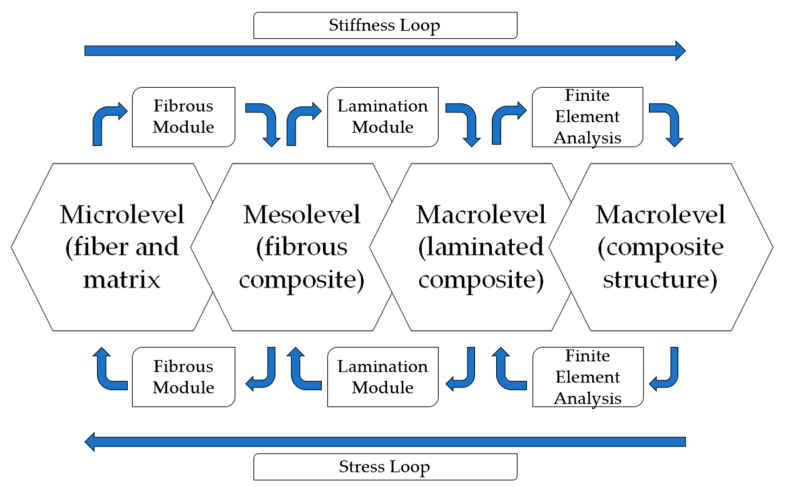
Hierarchy of multiscale analysis for a unidirectional fiber reinforced composite.

**Figure 7:**
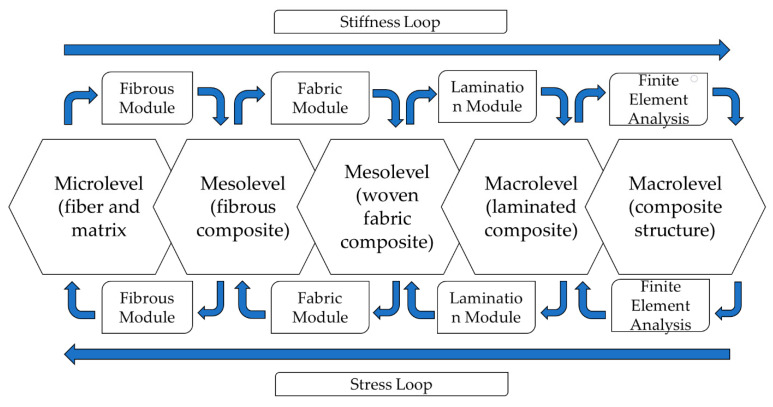
Multiscale analysis hierarchy for a two-way fiber reinforced composite.

Kwon, Allen and Talreja [47] created flowcharts (Figure 6 and Figure 7) exemplifying what happens at each of the scales, where the microscale basically approaches the individual fiber and matrix–fiber interfaces, the mesoscale approaches the individual layers and the macroscale considers effect of the complete laminate homogeneously [48]. 

Initially, Kwon, Allen and Talreja [47] developed the flowchart for unidirectional composites (UD) (Figure 6). First, in the "Fiber Module," the properties of the fiber and matrix materials, and the geometric properties, are correlated to defining the composite properties. These properties are used for each blade with its fiber orientation relative to the global coordinate system [47]. 

The "Laminating Module" calculates the properties of the laminated composite, so these properties are used for finite element analysis of the structure, completing the stiffness loop. The inverse order is used to decompose the stress and deformations from the macro level to the micro-level; i.e., stress and deformations in the fiber and matrix materials. 

After the calculation of microscale stresses and strains, the damage and/or failure criteria are applied. Because damage and failure are described at the constituent level, damage and failure modes are simplified and based on physics. At the microscale, there are three possible damages and failures: fiber rupture, matrix failure and interface detachment. Different failure or damage criteria can be applied to these three damage modes. At the macro level, there are more complex damage modes, such as delamination. Only the location and orientation of the damage or failure will dictate the difference between the macroscale failure modes. As a result, the damage and failure modes can be understood in unified and simplified concepts [47].

For the woven fibers (MD), the "Fabric Module" is added which relates the properties of the UD fiber to the effective properties of the fabric (Figure 7). The purpose of this module is to calculate the properties of the fabric using information from the UD fibers and woven fabric and the decomposition of the deformations and tensions in the fibers [47].

**Figure 8:**
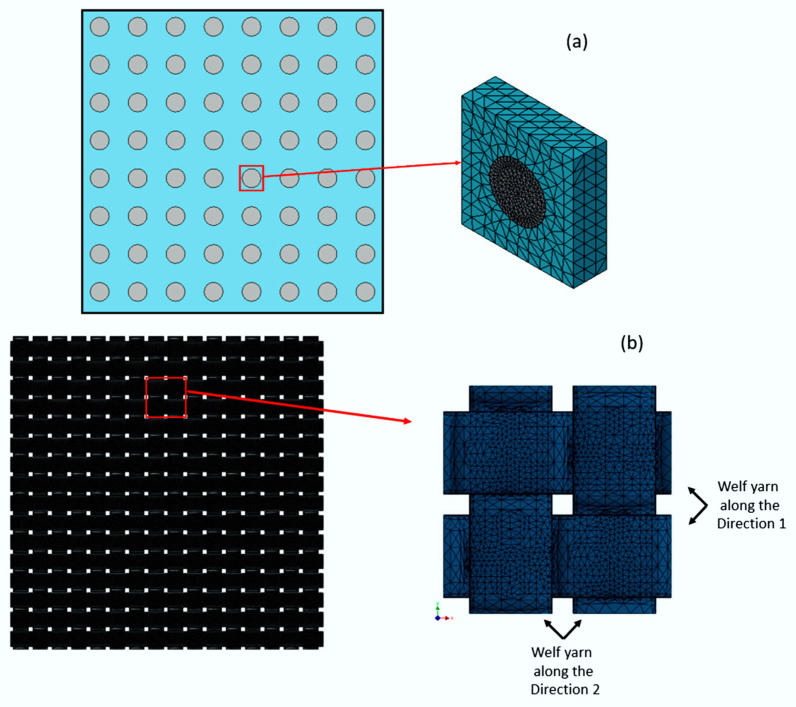
(**a**) Square arrangement of the microscale unit cell and (**b**) mesoscale, macroscopic unit cell.

Mao et al. [43] demonstrates in his work how to apply this methodology. The authors started the modelling from the micromechanical computation (Figure 8a), for which a unit cell with a square arrangement was used to represent the behavior of the UD material of the fiber yarn.

It is assumed that the fibers are arranged in a uniform distribution with the measured volumetric fraction and the same average filament diameter. In addition, the mesoscale unit cell (Figure 8b) is modelled to describe the woven architecture of the fiber yarns and the composite resin pocket. Two types of fiber yarn are modelled: weft yarn (longitudinal direction) and warp yarn (transverse direction). In FE analysis, periodic boundary conditions are used to eliminate boundary effects.

**Figure 9:**
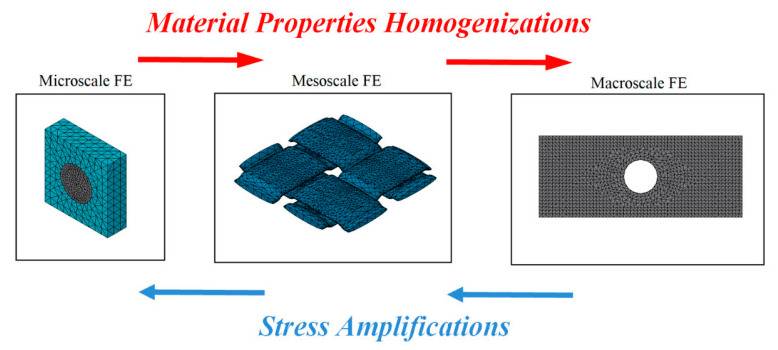
Multiscale modelling strategy for woven composite laminates.

As shown in Figure 9, the strained material properties of the macroscale model are obtained from micromechanical and mesomechanical analyses.

According Tian et al. [49], mechanical behaviors of heterogeneous materials are often described by using RVEs in the FE. The author mentioned two theories for RVE: first, Hill’s theory, for which the RVE must be large enough to contain a large number of fibers in the heterogeneous materials and be a statistical representation of the heterogeneous materials. The effective properties derived from the RVEs represent the real properties of the material on the macroscopic scale, which is commonly known as the micro-meso-macro principle, since scale separation is necessary [50]. Alternatively, in Drugan and Willis’s theory, the RVE must contain the smallest volume of composites for which the mean mechanical responses remain constitutively valid [51].

Tian et al. [49] pointed out that FE with RVEs is not common for modeling composites reinforced by fibers randomly distributed on a microscale, because these micro-architectures are much more difficult to model sometimes due to the high-volume fractions and large fiber aspect ratios to consider [52,53]. Therefore, in order to numerically model composites reinforced by spatially randomly discontinued fibers, it is important to generate RVEs with high fiber volume fractions and large fiber proportions.

The literature mentions two approaches as the most usual ones, namely, the random sequential adsorption (RSA) algorithm and the Monte Carlo (MC) procedure, for generating the artificial RVEs with randomly distributed fibers [49,51,52,53]. However, according to Lu, Yuan and Liu [50], with these approaches it is difficult to generate RVEs with high fiber aspect ratios (FARs) and fiber volume fractions (FVFs). A new approach to try to circumvent this situation is the use of the automatic searching and coupling (ASC) technique, in which it is possible to generate the 3D RVE to analyze the composite with random fibers with a wide range of FARs [50,51,54].

**Figure 10:**
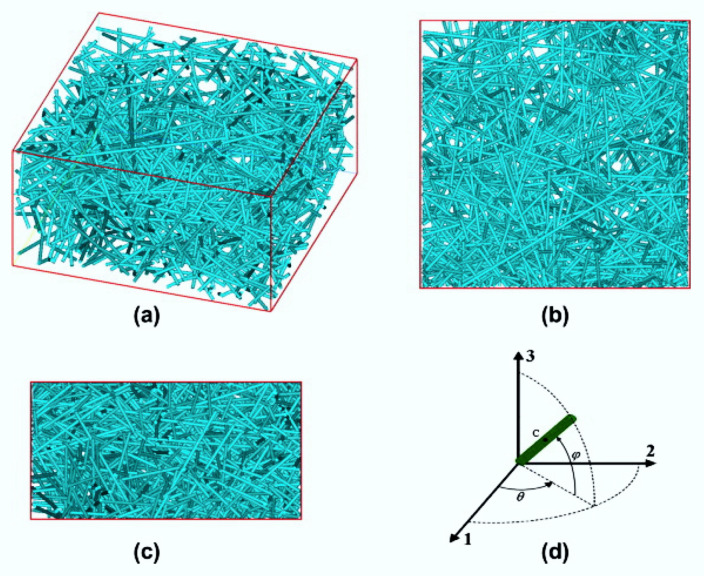
The sketch for the fiber architectures in the 3D model, (**a**) overall spatial view, (**b**) top vie, (**c**) side view, and (**d**) a fiber is described by the center point C, and two Euler angles θ and φ. Reproduced with permission [50].

Lu, Yuan and Liu [50] say: “compared with the conventional model, the present model is easier to generate and more time-saving as it eliminates the drawback of free meshing. In addition, the ASC technique can remove the additional stiffness introduced by the embedded element technique, and hence can improve precision and convergence. Moreover, our technique facilitates the direct application of the 3D periodic boundary conditions to the RVE.” See Figure 10.

### Cohesive Zone Model

**Figure 11:**
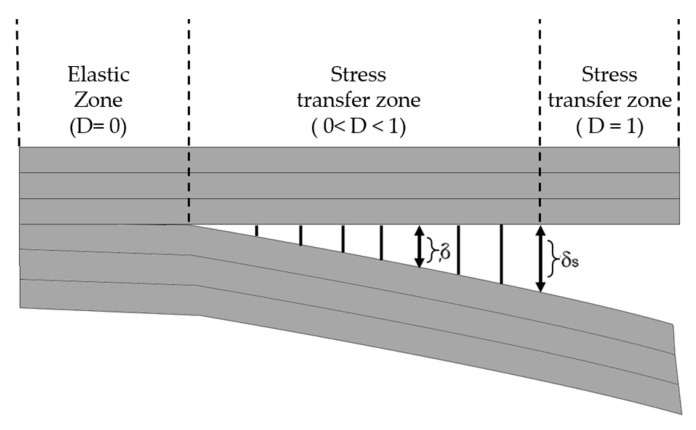
Cohesive zone model to simulate crack propagation.

Another method that has been widely applied in composite materials is the cohesive zone model (CZM). According to Barbero [27] the CZM is based on the assumption that the stress transfer capacity between the two separating faces of delamination is not lost completely at damage initiation, but rather is a progressive event governed by progressive stiffness reduction of the interface between the two separating faces (Figure 11). 

The models are typically expressed as a function of normal and tangential tractions in terms of separation distances. The forms of the functions and parameters change from model to model [55,56,57,58]. 

CZM has been used previously to study crack tip plasticity and creep under static and fatigue loading conditions, and in polymer cracks, adhesive joints, interface cracks in biomaterials and crack bridging due to fibers and ductile particles in composites [55,58,59]. Currently, CZMs are increasingly being used to simulate discrete fracture processes in various systems of homogeneous and non-homogeneous materials [55,56,57,60], with great emphasis on understanding the evolution of delamination in laminates [55,59,61,62,63,64,65].

According to Roy [58], in the presence of a large fracture process zone near the crack tip, the basic assumptions of the mechanics of linear elastic fracture are no longer valid. Specifically, in some polymers, such as hardened epoxies, the occurrence of void nucleation and growth ahead of the crack tip results in a damage zone that is not free from traction. Additionally, for a crack in a composite with a fiber-reinforced polymer matrix, the fiber bridge may be present within the damage zone. Therefore, in these cases, a cohesive layer modelling approach would be more accurate at accounting for non-linear processes that occur within the "damage zone" [58]. Furthermore, the interface modelling using CZM has a distinct advantage compared to other global approaches (e.g., shear lag model), in that it is based on a micromechanical method [55].

The cohesive zone model was proposed by Barenblatt in the 1960s based on Griffith’s theory of fracturing, in order to investigate the crack propagation in brittle materials. He assumed that finite molecular cohesion forces exist near the crack faces and described the crack propagation in perfectly brittle materials using his model. Then, Dugdale extended this concept to the perfectly plastic materials by postulating the existence of a process zone at the crack tip [55,57,58,66,67]. Roy [58] mentions that in the coming years, many uses of CZMs were used to better understand the functioning of cracking in laminated composites, with the objective of capturing the Burridge-Andrew mechanism using the material point method.

Using the cohesive modelling, no additional properties are necessary to simulate crack growth. Only the cohesive law is needed to analyze both the initiation and growth of a crack. Typically, cohesive elements in FEM codes follow a predefined traction separation law that simulates the crack initiation and propagation. Another advantage of CZMs is that these models can simulate different types of failure mechanisms, such as fiber-matrix debonding and interlaminar delamination [58,61,62,65,68,69].

Chaboche [57] mentions that the cohesive model considers the presence of a process zone at the tip of the crack, with an appropriate constitutive law, relating the normal tensile stress (T) and the relative displacement (u) between the two sides of the crack. The relation between T and u is characterized by a softening law (decreasing function) and the area under the stress-displacement response corresponds to the fracture energy Gc of the material.

**Figure 12:**
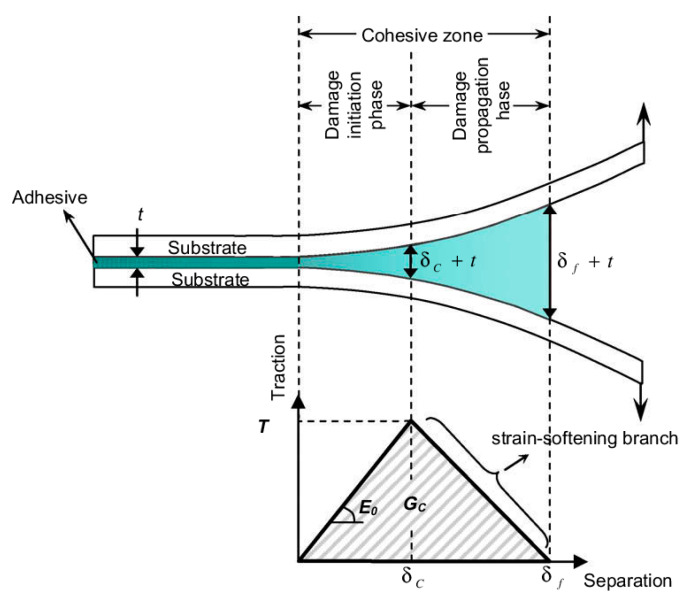
Schematic damage process zone and corresponding bi-linear traction–separation law in an adhesively bonded joint. Reproduced with permission [67].

According to Khoramishad et al. [67] the cohesive zone model (Figure 12), combines a strength-based failure criterion to predict the damage initiation and a fracture mechanics-based criterion to determine the damage propagation. 

## 3. Constitutive Laws of a Composite Material

In general, the constitutive equation of a linear elastic solid is known as the generalized Hooke’s Law, which relates nine Cauchy stress components with nine deformation components, giving a total of eighty-one constants [31,70,71,72]. 

Equations (1) and (2) present the combination of elasticity constants, where $\sigma_{ij}$ represents the stress component, $\varepsilon_{kl}$ the strain components, $Q_{ijkl}$ the stiffness matrix and $S_{ijkl}$ the compliance matrix of the material, those being inversely related as follows: $\left[S\right]={\left[Q\right]}^{-1}$.

(1) $$\sigma_{\mathrm{ij}}=Q_{\mathrm{ijkl}}{\;\varepsilon }_{\mathrm{kl}}$$

(2) $$\varepsilon_{\mathrm{kl}}=S_{\mathrm{ijkl}}{\;\sigma }_{\mathrm{ij}}$$

According to Bednarcyk, Aboudi and Arnol [73], and Bauchau and Craig [74] the physical phenomena such as heat conduction, diffusion, electric permittivity, magnetic permeability and electric conductivity are governed by the material constitutive laws. These constitutive laws characterize the mechanical behavior of a material and consist of a set of mathematical idealizations of the observed behavior [74].

### 3.1. Anisotropic Material

Due to the growing importance of composite materials, the linearly elastic behavior of anisotropic materials must be understood. The physical properties of anisotropic materials are directional; i.e., the physical response of the material depends on the direction in which it acts [74].

According to Soriano [75] and Daniel and Ishai [72], a linear anisotropic material has a matrix with independent elastic properties, which, in turn, make the characterization very difficult. In general, the stiffness matrix has 36 independent coefficients (Equation (3)), owed to the symmetry between $\sigma_{ij}$ and $\sigma_{ji}$, and between $\varepsilon_{kl}$ and $\varepsilon_{lk}$; hence the reduction from 81 to 36 elastic constants [3,31,70].

(3) $$\left\{\begin{array}{c} \sigma_{11}\\ \sigma_{22}\\ \sigma_{33}\\ \sigma_{23}\\ \sigma_{13}\\ \sigma_{12} \end{array}\right\}=\left[\begin{array}{cccccc} Q_{11} & Q_{12} & Q_{13} & Q_{14} & Q_{15} & Q_{16}\\ Q_{21} & Q_{22} & Q_{23} & Q_{24} & Q_{25} & Q_{26}\\ Q_{31} & Q_{32} & Q_{33} & Q_{34} & Q_{35} & Q_{36}\\ Q_{41} & Q_{42} & Q_{43} & Q_{44} & Q_{45} & Q_{46}\\ Q_{51} & Q_{52} & Q_{53} & Q_{54} & Q_{55} & Q_{56}\\ Q_{61} & Q_{62} & Q_{63} & Q_{64} & Q_{65} & Q_{66} \end{array}\right]\left\{\begin{array}{c} \begin{array}{c} \varepsilon_{11}\\ \varepsilon_{22} \end{array}\\ \varepsilon_{33}\\ 2\varepsilon_{23}\\ 2\varepsilon_{13}\\ 2\varepsilon_{12} \end{array}\right\}$$

However, the symmetry requirement for anisotropic materials is reducing the elastic components to 21, by the relation $Q_{ij}=Q_{ji}$ [3,31,70,74]. Azevedo [71] pointed out that elastic properties required to define an anisotropic material can be represented by stress–strain ratios, the main coefficients being: longitudinal and transverse moduli of elasticity and the Poisson’s coefficient. Equation (4) shows the generalised mathematical representation of Hooke’s law for anisotropic materials, where E is the longitudinal elasticity modulus or Young’s modulus, G is the transverse modulus of elasticity or shear modulus, υ is the Poisson’s coefficient and ρ is the angular/linear deformation.

(4) $$\left\{\begin{array}{c} \begin{array}{c} \varepsilon_{11}\\ \varepsilon_{22} \end{array}\\ \varepsilon_{33}\\ 2\varepsilon_{23}\\ 2\varepsilon_{13}\\ 2\varepsilon_{12} \end{array}\right\}=\left[\begin{array}{cccccc} \;\frac{1}{E_{1}} & -\frac{\upsilon_{21}}{E_{2}} & -\frac{\upsilon_{31}}{E_{3}} & \frac{\rho_{2311}}{G_{23}} & \frac{\rho_{1311}}{G_{13}} & \frac{\rho_{1211}}{G_{12}}\\ -\frac{\upsilon_{12}}{E_{1}} & \;\frac{1}{E_{2}} & -\frac{\upsilon_{32}}{E_{3}} & \frac{\rho_{2322}}{G_{23}} & \frac{\rho_{1322}}{G_{13}} & \frac{\rho_{1222}}{G_{12}}\\ -\frac{\upsilon_{13}}{E_{1}} & -\frac{\upsilon_{23}}{E_{2}} & \;\frac{1}{E_{3}} & \frac{\rho_{2333}}{G_{23}} & \frac{\rho_{1333}}{G_{13}} & \frac{\rho_{1233}}{G_{12}}\\ \frac{\rho_{1123}}{E_{1}} & \frac{\rho_{2223}}{E_{2}} & \frac{\rho_{3323}}{E_{3}} & \frac{1}{G_{23}} & \frac{\rho_{1323}}{G_{13}} & \frac{\rho_{1223}}{G_{12}}\\ \frac{\rho_{1113}}{E_{1}} & \frac{\rho_{2213}}{E_{2}} & \frac{\rho_{3313}}{E_{3}} & \frac{\rho_{2313}}{G_{23}} & \frac{1}{G_{13}} & \frac{\rho_{1213}}{G_{12}}\\ \frac{\rho_{1112}}{E_{1}} & \frac{\rho_{2212}}{E_{2}} & \frac{\rho_{3312}}{E_{3}} & \frac{\rho_{2312}}{G_{23}} & \frac{\rho_{1312}}{G_{13}} & \frac{1}{G_{12}} \end{array}\right]\left\{\begin{array}{c} \sigma_{11}\\ \sigma_{22}\\ \sigma_{33}\\ \sigma_{23}\\ \sigma_{13}\\ \sigma_{12} \end{array}\right\}$$

For Vanalli [76] the failure analysis of structures made out of anisotropic materials is complex. The author emphasized that in these cases it must be assumed that the failure is caused by normal and shear stresses, since failure can occur due to different sets of stress acting on the element.

### 3.2. Orthotropic Material

Comparing an orthotropic material with a generally isotropic theory, it is observed that the first one presents three symmetry planes orthogonal to each other—x_1_ x_2_, x_1_ x_3_ e x_2_ x_3_, thereby reducing from 21 to 9 the number of independent coefficients [3,31,70,71,72,77]. The reasons for that are:

➢ The angular deformations are independent of normal stress;
➢ Linear deformations are independent of tangential stresses;
➢ Each tangential tension causes only angular deformation in the plane in which it acts.

Equation (5) shows the generalised representation by Hooke’s law for orthotropic materials, where E is the modulus of longitudinal elasticity or Young’s modulus, G is the cross modulus of elasticity or shear modulus and υ is the Poisson coefficient [71].

(5) $$\left\{\begin{array}{c} \begin{array}{c} \varepsilon_{11}\\ \varepsilon_{22} \end{array}\\ \varepsilon_{33}\\ 2\varepsilon_{23}\\ 2\varepsilon_{13}\\ 2\varepsilon_{12} \end{array}\right\}=\left[\begin{array}{cccccc} \;\frac{1}{E_{1}} & -\frac{\upsilon_{21}}{E_{2}} & -\frac{\upsilon_{31}}{E_{3}} & 0 & 0 & 0\\ -\frac{\upsilon_{12}}{E_{1}} & \frac{1}{E_{2}} & -\frac{\upsilon_{32}}{E_{3}} & 0 & 0 & 0\\ -\frac{\upsilon_{13}}{E_{1}} & -\frac{\upsilon_{23}}{E_{2}} & \frac{1}{E_{3}} & 0 & 0 & 0\\ 0 & 0 & 0 & \frac{1}{G_{23}} & 0 & 0\\ 0 & 0 & 0 & 0 & \frac{1}{G_{13}} & 0\\ 0 & 0 & 0 & 0 & 0 & \frac{1}{G_{12}} \end{array}\right]\left\{\begin{array}{c} \sigma_{11}\\ \sigma_{22}\\ \sigma_{33}\\ \sigma_{23}\\ \sigma_{13}\\ \sigma_{12} \end{array}\right\}$$

When considering a composite reinforced with unidirectional fibers, it would be automatically classified as an anisotropic material because there is no symmetry between the planes. If we analyse the thickness, we can see that it is much smaller in size than any other plane dimension. Because of this, many researchers consider unidirectional laminates as orthotropic materials, taking into account the plane stress state, according to the hypotheses shown in Equation (6) [70].

(6) $$\sigma_{3}=\tau_{23}=\tau_{31}=0$$

### 3.3. Transverse Isotropic Material

A transverse isotropic material can be defined as an orthotropic material that presents isotropy in one of the planes of symmetry, which means it has the same properties in all directions in this plane [31,71,72,74].

Comparing a transverse isotropic material with a generally orthotropic material, it is observed that in transverse isotropic materials there is a symmetry between the planes x_1_ x_3_ and x_1_ x_2_, reducing nine to five independent coefficients. According to Azevedo [71] beyond the considerations for orthotropic materials, it can be added:

➢ The linear deformations in the plane x_2_ x_3_ caused by the normal stress σ_11_ are equal;
➢ The linear deformations ε_22_ and ε_33_ caused by the normal stress σ_22_ are equal to the deformations ε_33_ and ε _22_, respectively, caused by a tension σ_22_ = σ_33_;
➢ Each tangential tension only causes angular deformation in the plane in which it acts;
➢ The angular strain γ_23_ caused by a stress σ _23_ is equal to an angular strain γ_13_ caused by stress σ_13_ = σ_23_.

Equation (7) presents the generalised representation by Hooke’s law for transverse isotropic material, where E is the modulus of longitudinal elasticity or Young’s modulus, G is the transverse modulus of elasticity or shear modulus and υ is the Poisson coefficient [71].

(7) $$\left\{\begin{array}{c} \begin{array}{c} \varepsilon_{11}\\ \varepsilon_{22} \end{array}\\ \varepsilon_{33}\\ 2\varepsilon_{23}\\ 2\varepsilon_{13}\\ 2\varepsilon_{12} \end{array}\right\}=\left[\begin{array}{cccccc} \;\;\frac{1}{E_{1}} & -\frac{\upsilon_{21}}{E_{2}} & -\frac{\upsilon_{13}}{E_{1}} & 0 & 0 & 0\\ -\frac{\upsilon_{12}}{E_{1}} & \frac{1}{E_{2}} & -\frac{\upsilon_{12}}{E_{1}} & 0 & 0 & 0\\ -\frac{\upsilon_{13}}{E_{1}} & -\frac{\upsilon_{21}}{E_{2}} & \frac{1}{E_{1}} & 0 & 0 & 0\\ 0 & 0 & 0 & \frac{1}{G_{12}} & 0 & 0\\ 0 & 0 & 0 & 0 & \frac{1}{G_{13}} & 0\\ 0 & 0 & 0 & 0 & 0 & \frac{1}{G_{12}} \end{array}\right]\left\{\begin{array}{c} \sigma_{11}\\ \sigma_{22}\\ \sigma_{33}\\ \sigma_{23}\\ \sigma_{13}\\ \sigma_{12} \end{array}\right\}$$

## 4. Failure Criteria

According to Kaw [3] the success when using a composite structure is related to its efficiency and safety. For this, some criteria were adopted to identify possible failures associated with a component. For Ochoa and Reddy [78] and Jones [31] and Kaw [3], a failure criterion aims to provide a comprehension of the effects caused by combined loads (double or triple stress state) in the structure, indicating when there is a local or global failure. For Kaw [3], in general, the theories are related to normal and shear forces of the laminate, defining the stress states in which the failure occurs.

According to Jones [31] and Kaw [3], failure criteria were initially created for isotropic materials, where maximum normal and shear stresses of the material were found when the maximum stress was greater than the last force, indicative of material failure.

**Figure 13:**
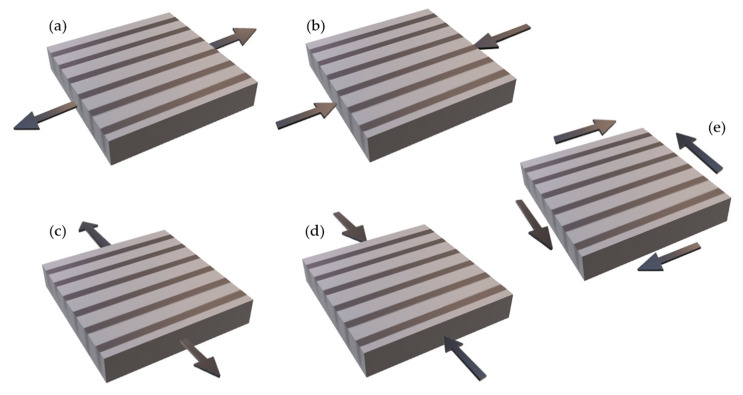
Basic strength parameters of unidirectional lamina for in-plane loading, (**a**) longitudinal tensile, (**b**) longitudinal compressive, (**c**) transverse tensile, (**d**) transverse compressive, and (**e**) in-plane or interlaminar shear.

Among many failure criteria relevant to isotropic materials are the maximum normal stress (Rankine), maximum shear stress (Tresca), maximum normal strain (Saint–Venant) and maximum strain theory (Von Mises) [3,31,72,79]. Feng [80] and Kaw [3] cite that based on these theories, the failure criteria for anisotropic and orthotropic materials were developed; based on the orientation of the fibers, four parameters of normal resistance and one of shear resistance are considered, making a total of five fundamental resistance parameters for the use of the failure criterion (Figure 13).

The failure theory problem of an orthotropic sheet to a certain extent is identical to the isotropic one, which in this case is the prediction of when a sheet is submitted to a biaxial or triaxial stress state, using resistance data obtained from uniaxial experiments [81]. 

Currently, there are several failure criteria for composites: Hill, Tsai–Hill, Tsai–Wu, Hashin-Rotem, Hashin, maximum stress, Hoffman, maximum strain, Hou, Puck–Schürmann, Chang-Chang, Linde, LaRC03, LaRC04, Maimí, Hart-Smith, Yeh-Stratton and others [16,82,83,84,85,86,87,88]. 

According to Mendonça [81], some of these criteria are of common use and well-established in the literature. Normally, these criteria are characterized by ignoring any aspect from any physical process involved in the failure, considering only macroscopic effects observed in the standard specimen. The most widely-used failure criteria are the maximum stress, Tsai–Hill, Tsai–Wu, Hashin and Puck–Schürmann (Table 1).

The maximum stress criterion, based on Rankine’s theory, is not an interactive criterion; i.e., it does not consider the combined effects of the various components of the tensor. This criterion provides for the rupture when one of the tensor components arrives at the corresponding tensile stress [179,180]. 

Hill, in 1950, based on the von Mises criterion, established one of the first failure criteria for anisotropic materials, which is a generalisation of the flow behavior for isotropic materials [181]. Although it is a more general criterion, it has as a drawback: the determination of several parameters to establish the complete equation of the model. In 1965, Tsai proposed a modified Hill criterion where he quantified the traction and compression inequality for orthotropic materials, which was called the Tsai–Hill criterion [181]. For several authors, the Tsai–Hill criterion is one of the best and most widely used failure criteria for laminates because it considers the interactions between the stress components; however, this criterion is not invariant in relation to the coordinate system; therefore, only orthotropic materials should be applied [3,31,81,179,182,183]. Despite being considered one of the best failure criteria, many consider that this criterion has several deficiencies in its theoretical basis [81,179]. Mendonça (2005) cites that there are basically three deficiencies in Tsai–Hill’s theory; namely:

➢ It does not intrinsically consider differences in tensile and compressive strength;
➢ It does not present good results in the state of loading by compression in the three main axes;
➢ It supposes that a hydrostatic state of stresses cannot cause failure—in the case of anisotropic materials, a hydrostatic state of stress causes shear deformation and failure.

Tsai and Wu in 1971 presented a criterion based on the Tsai–Hill criterion, aiming to increase the number of terms in the Tsai–Hill failure criterion equation, to better approximate the experimental data, considering a two-dimensional stress state [81,181,182]. The Tsai–Wu criterion is an interactive criterion, which provides for component rupture due to the combination of tensions acting on the part [179,180,183]. In addition, this criterion, in its three-dimensional form, takes into account the effect of the hydrostatic component of the stresses differently from the previously-described criteria [184]. The interactions between the stress components are independent of the material properties. However, since it is not a failure criterion based on physical phenomena, it can predict the occurrence of the damage, but cannot distinguish between the different failure modes; it can only predict whether or not the failure occurs in the structure [81,180,183,184]. The Tsai–Wu criterion became one of the most used criteria, and to this day several works are developed based on the same. [181].

Hashin, in 1980, proposed a failure criterion divided into subcriteria, for failure in unidirectional fiber reinforced sheets—transversely isotropic—based on the quadratic polynomial of tensions [81,149,150,180,181,182,185]. Differently from the Tsai–Hill and Tsai–Wu criteria, which do not allow an identification of failure modes; the Hashin criterion considers modes of failure of the fiber and matrix, distinguishing between tensile and compression loads, addressing four main modes: traction and compression of the fibers and matrix [81,183,185]. According to Laurin, Carrere and Maire [186], the historical importance of this criterion is that it started a different way of designing failure criteria for composite materials. Hashin [150] first identified the predominant failure modes, and subsequently the variables associated with these modes, and then proposed the interactions between the variables involved in each failure mode. Despite the wide use of failure criteria, they present many difficulties regarding the accuracy of the results, because of undesired failure modes; plastic deformations and geometric nonlinearity of the parts; the effect of the residual tensions of the composite fabrication; and dispersions in the experimental results due to the heterogeneous nature of the materials [179].

Puck followed the failure theory framework of Hashin and proposed an elaborate scheme for implementation of his theory in Puck and Schürmann. As in Hashin, Puck’s theory (Puck, and Puck and Schürmann) recognizes a failure in UD composites to be in fiber failure (FF) and inter-fibre failure (IFF) modes [167,187,188,189]. For fiber failure, there are two modes of compression and traction. In the case of inter-fiber failure, there are Modes A, B and C, which include matrix fracture or fiber-matrix displacement. The inter-fiber failure modes were based on the Coulomb–Mohr’s fracture hypothesis which is appropriate for brittle fracture behavior of composite materials, wherein failure on a plane occurs when certain resistances, related to its cohesion and internal friction, are overcome [93,164,187,190]. Acoording to Wang and Zhao [165], Puck’s criterion has become a mainstream failure criterion for predicting responses of a composite subjected to impact loads.

**Figure 14:**
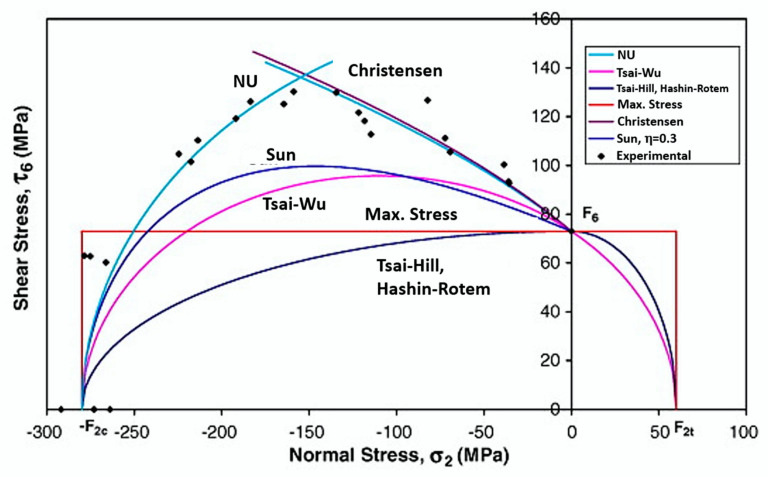
Comparison of theoretical and experimental results. Reproduced with permission [191].

**Figure 15:**
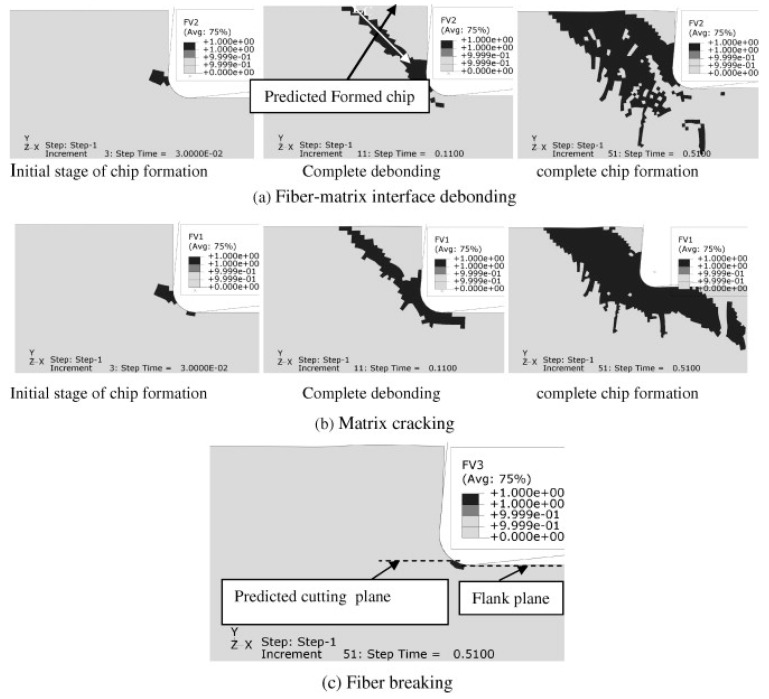
Progressive failure analysis with Hashin failure criteria for 45° fiber orientation. (**a**) Fiber–matrix interface detachment, (**b**) matrix fracture and (**c**) fiber rupture. Reproduced with permission [24].

Failure criteria are used to determine when material failure will occur. This concept can be applied in several areas, such as for mechanical tests, manufacturing processes, corrosive environments, etc.; for mechanical tests (Figure 14) and machining processes (Figure 15), they are the most used ones, where the criterion is combined with the material property degradation rule for the failure analysis.

## 5. Types of Elements Applied in Composite Modelling

For the analysis of a component, it is necessary to create a mesh on it [192]. The mesh is composed of elements and nodes. While the elements are subdivisions of the analyzed structure, the nodes are the connections of these subdivisions [21,193]. There are several types of elements; for example: bar, beam, columnar, triangular, quadrilateral, plate, shell, solid, etc. [21,194,195]. However, regarding an efficient and effective analysis for composite materials, there are four types mostly chosen: solids, beam, plate and shell.

Solid elements are the least used one for composites, because they require a model with many layers or a costly and time-consuming full-size structure, becoming consequently, unfeasible [196]. Besides these reasons, if the laminate thickness is very thin, layers constructed with solid elements can result in ill-conditioned equations. These factors lead to the use of other elements with lower computational demand and well-conditioned equations.

The beam element can be defined as having one of its dimensions larger than the others. One of the axes is defined along the longer dimension, and a cross-section taken perpendicular to this axis is assumed to vary smoothly along the beam length [74].

According to Bauchau and Craig [74] civil engineering structures often consist of an assembly or grid of beams with T or I-shaped cross-sections. A large number of machine parts are beam-like structures as well: lever arms, shafts, etc. Finally, several aeronautical structures such as wings and fuselages can also be treated as thin-walled beams.

Bauchau and Craig [74] cited that long and slender aircraft wings can be analyzed, as a first approximation, like beam structures, but a more refined and detailed analysis should treat separately the upper and lower skins of the wings as thin plates or shells supported by ribs and longerons, or stiffeners. Nevertheless, aircraft wings with small aspect ratios cannot be treated as beams because two their dimensions are larger than their thicknesses. But most of the time they can often be represented as plates. The aircraft fuselage is also constructed of thin-walled structures stiffened with ribs and longerons, and the thin-walled portions between the stiffeners can be drawn as thin plates. Last but not least, thin-walled beams can be modelled as plates when considering a localized behavior induced by attachments or supports.

Both plate and shell are considered two-dimensional or surface elements because two their 2D dimensions (length and width) are much larger than their thicknesses, which are given by the number of layers in their laminates [21,22,27,75,77,196,197,198,199,200,201]. Because of this, even with classical theory mathematically differentiating these two elements, the terms plate and shell are often used interchangeably, assuming that a plate element is flat, but when curved, it would become a shell element [21,74,197,198]. 

According to Barbero [77] whenever the thickness coordinates are eliminated from the general equation, they create a 3D problem in a 2D design. The author even mentioned that modelling laminate composites differ from any conventional materials modelling in three aspects:

➢ The constitutive equations of each layer are orthotropic;
➢ The constitutive equations of the element depend on the kinematic considerations of the plate/shell theory employed and its implementation on the element;
➢ The symmetry of the material is as important as the geometry and symmetry of the loading when trying to use conditions of symmetry in the models.

Regarding structural composites, plate and shell elements are the most used types [74,202]. However, according to Tenek and Argyris [22] the use of a 2D element may neglect the flexural stiffness of a material. This issue does not exist in solid elements once they are a 3D type of structure; i.e., the thickness is included in the general equation.

### 5.1. Plate Element

A plate is a 2D plane solid element whose thickness (h or t), usually measured in the z-axis direction, is a lot smaller than its length and width, which are located in the *xy* plane [21,203,204,205]. The plate element can support actions that promote transverse flexion; in addition, it has two bending moments and one torsional moment [21,75,196,201].

The most classic examples of plates are slabs; buildings’ floor slabs; bridge decks; sides of rectangular water tanks and other fluid retaining structures; and tables. They transmit the loads that act on the normal direction of their midplane (z-axis) [75,199,201]. The two main theories that describe a plate element behavior are Kirchhoff theory and Mindlin theory, both being based on kinematic hypotheses [21,75,196,201,206,207,208,209].

#### 5.1.1. Elements of Kirchhoff Theory

**Figure 16:**
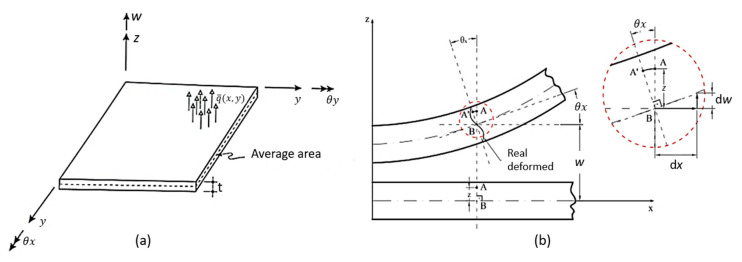
(**a**) Section of plate of thickness t, under transverse loading $\bar{q}$ per unit area, where w is a transverse displacement of a point of the mean surface, and $\theta_{x}\;\mathrm{and}\;\theta_{y}$ are the rotations normal to the same point according to the x and y axes. Reproduced with permission [75]. (**b**) Field of displacements according to Kirchhoff’s plate theory. Reproduced with permission [212].

Classic Kirchhoff theory, also called classical theory, is analogous to Euler–Bernoulli’s beam theory; it is employed in the study of thin plates [74], whose relationship between the smallest plate gap and the thickness (t) is less than twenty [75,196,201,208]. This theory considers the thickness to be inextensible and neglects the shear strain deformations, assuming that a normal line segment at the mean surface remains rectilinear and perpendicular to the surface after deforming the plate (Figure 16) [75,206,208,210,211]. Altenbach and Eremeyev [203] added that Kirchhoff theory considers that the plate is made of a homogeneous, isotropic, linear elastic material. It assumes the validity of generalized Hooke’s law. According to Vaz [201], Altenbach and Eremeyev [203], Saliba et al. [212] and Schneider et al. [213], the kinematic hypotheses of Kirchhoff’s theory for plates with total isotropy are:

➢ Any point P (x, y) on the average surface of the plate moves only in the z direction—that is, it has only vertical displacement w (x, y);
➢ The normal stress in the z-direction (σz) is negligible;
➢ The longitudinal strain is zero at any point on the plate, i.e., $\varepsilon_{z}$ = 0;
➢ A straight and normal line to the average surface before loading and cutting the median plane of the plate at point P (x, y) remains straight and normal to the plane tangent to the average surface at that point after loading, and therefore, the shear deformations $\gamma_{yz}$ e $\gamma_{xz}$ are zero.

Based on the third hypothesis above, it is possible to define expressions that describe the displacement fields of the plates, which are represented in Equation (8) [199,201].

(8) $$\{\begin{array}{c} u\left(x,y,z\right)=-z\frac{\partial w\left(x,y\right)}{\partial x}\\ v\left(x,y,z\right)=-z\frac{\partial w\left(x,y\right)}{\partial y} \end{array}$$

According to Vaz [201] it is possible to obtain deformations for an infinitesimal plane element from z-dimension parallel to the mean plane of the plate (Equation (9)), ε being the deformation vector of a given point of the plate, and $k_{k}$ is the vector containing the curvatures of Kirchhoff’s theory relative to a point in the middle plane of the plate which is on the same vertical line as the point where ε was calculated.

(9) $$\left\{\begin{array}{c} \varepsilon_{x}\\ \varepsilon_{y}\\ \gamma_{xy} \end{array}\right\}=-z\left\{\begin{array}{c} w_{xx}\\ w_{yy}\\ 2w_{xy} \end{array}\right\}$$

Vaz [201] also pointed out that for plate element, there are actions from bending moments and shearing forces, and the shear moment can lead to vertical shear stresses, and consequently, distortions $\gamma_{yz}$ and $\gamma_{xz}$, which are zero, as observed in Equation (10). That is the reason why this theory can only be applied to thin plates.

(10) $$\{\begin{array}{c} \gamma_{xz}=\frac{\partial w}{\partial x}+\frac{\partial u}{\partial z}=w_{y}-w_{y}=0\\ \gamma_{yz}=\frac{\partial w}{\partial y}+\frac{\partial v}{\partial z}=w_{x}-w_{x}=0 \end{array}$$

Soriano [75] and Vaz [201] cited that through the constitutive relations, it is possible to obtain the general equation for the element (Equations (11) and (12)).

(11) $$\left\{\begin{array}{c} M_{x}\\ M_{y}\\ M_{xy} \end{array}\right\}=D\left[\begin{array}{ccc} 1 & v & 0\\ v & 1 & 0\\ 0 & 0 & \frac{\left(1-v\right)}{2} \end{array}\right]\left\{\begin{array}{c} \begin{array}{c} w_{xx}\\ w_{yy} \end{array}\\ 2w_{xy} \end{array}\right\}$$

where:

(12) $$D=\frac{Et^{2}}{12\left(1-v^{2}\right)}$$

In Equation (13), the summarized form of the equation is given, where {M} is the vector of moments at a point on the middle surface of the plate, [E] is the plate bending stiffness matrix by Kirchhoff theory and {ε} is the deformation vector at a given point [75,201].

(13) {M}=[E]{ε}

Considering the previous equations, it is possible to obtain the deformation energy for an element (Equation (14)); the total potential energy (Equation (15)), where $\bar{q}$ is the transverse force per unit of positive area in the z-direction; and the general equilibrium equation or the minimum potential energy principle (Equation (16)) [22,75,202,204].

(14) $$U=\frac{1}{2}\int_{A}⎣w_{xx}\;w_{yy}\;2w_{xy}⎦\left\{\begin{array}{c} M_{x}\\ M_{y}\\ M_{xy} \end{array}\right\}dA$$

(15) $$\Pi =\frac{1}{2}\int_{A}⎣w_{xx}\;w_{yy}\;2w_{xy}⎦\left[E\right]\left\{\begin{array}{c} \begin{array}{c} w_{xx}\\ w_{yy} \end{array}\\ 2w_{xy} \end{array}\right\}dA-\int_{A}\bar{q}w\;dA$$

(16) $$\frac{\partial^{4}w}{\partial x^{4}}+\frac{2\partial^{4}w}{\partial x^{2\;}\partial y^{2}}+\frac{\partial^{4}w}{\partial y^{4}}=\frac{\bar{q}}{D}$$

**Figure 17:**
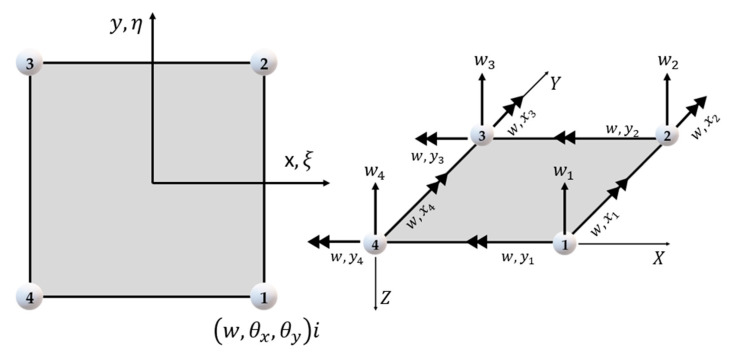
Rectangular plate element by Kirchhoff’s theory.

Through Kirchhoff theory, two elements were developed, those being the rectangular and triangular [75,199,201,212,214]. For rectangular elements there are two types; one element is nonconforming and has three degrees of freedom per node: a vertical displacement (w) and two rotations (θx and θy) (Figure 17). The other has four degrees of freedom per node: a displacement vertical (w), two rotations (θx and θy) and a curvature (w, xy) [75,212,215,216,217,218,219].

**Figure 18:**
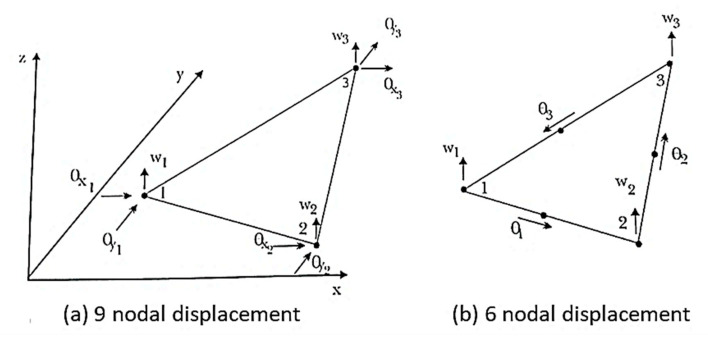
Non-conforming triangular elements. Reproduced with permission [75].

Soriano [75] cite that the rectangular element does not have a constant shear state. Because of this, Kirchhoff’s triangular element was created, and it can have six or nine nodal displacements. Saliba [214] and Saliba et al. [212] cite that Kirchhoff’s nonconforming triangular element with nine terms was developed by Cheung et al. in 1968 and that the element has the three degrees of freedom per node: a vertical displacement (w) and two rotations (θx and θy) (Figure 18a). Soriano [75] argues that Morley in 1971 developed the nonconforming but convergent Kirchhoff triangular element with six terms, which has only vertex transverse displacements and normal rotations at the midpoints of the sides (Figure 18b).

**Figure 19:**
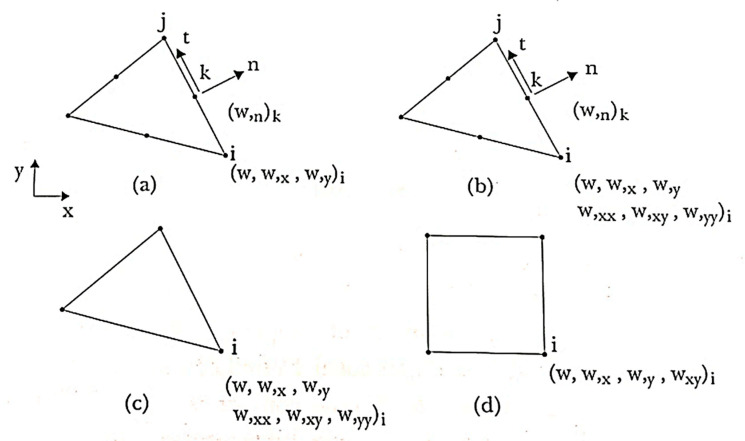
Compliant thin-plate elements. Reproduced with permission [75].

Soriano [75] and Saliba [214] report the existence of other elements with discrete constraints and conforming elements, those being three triangular and one rectangular element (Figure 19).

#### 5.1.2. Elements of Mindlin Theory

Mindlin or Reissner–Mindlin theory for plates is equivalent to Timoshenko theory for beams, in which the main hypothesis is that the cross section of the beam remains flat, but not necessarily perpendicular to the tangent of the elastic line when deformed [21,203,207,209,210,213]. Mindlin theory is hierarchically superior to the Kirchhoff because it presents a three-dimensional solution and can be applied for both thin plates and spines [75,206,207,208]. Mindlin theory is a shear-deformable plate theory [203].

According to Soriano [75], Altenbach and Eremeyev [203], Saliba et al. [212] and Vaz [201], the kinematic hypotheses of Mindlin’s theory are:

➢ Any point P (x, y) on the average surface of the plate moves only in the z direction—that is, it has only vertical displacement w (x, y);
➢ The normal stress in the z direction (σz) is negligible;
➢ The vertical longitudinal strain is zero at any point on the plate—i.e., εz = 0;
➢ A straight and normal line to the average surface before loading and cutting the median plane of the plate at point P (x, y), remains straight after loading, and straight but not necessarily normal to this plane, after deformation.

**Figure 20:**
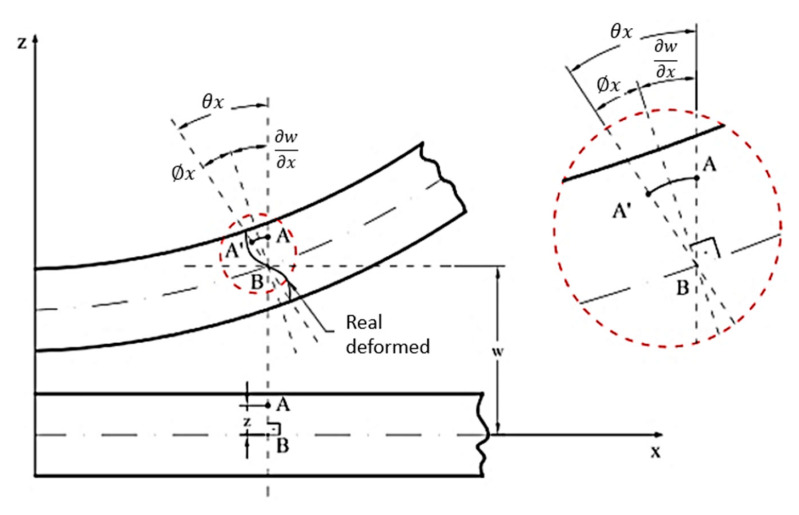
Field of displacements according to Reissner–Mindlin plate theory Reproduced with permission [212].

The change in the fourth hypothesis reflects in the displacement field of the plate (Figure 20), even though it does not link the rotation of the vertical line passing through P (x, y) to the derivatives of the vertical displacement w (x, y) [201,212,220].

According to Vaz [201], based on the third hypothesis, it is possible to define the expressions describes the plate displacement fields (Equation (17)).

(17) $$\{\begin{array}{c} u\left(x,y,z\right)=z\theta_{y}\\ v\left(x,y,z\right)=-z\theta_{x} \end{array}$$

Vaz [201] also mentioned that is possible to obtain the element deformation (Equation (18)), in which in the summarized equation, the deformation vector ε is subdivided into two vectors: $\varepsilon_{b}$ associated with the bending moments and $\varepsilon_{s}$ associated with the shear forces; $T_{M}$ is the transformation matrix subdivided into Tb and Ts; $k_{M}$ M is Mindlin curvature vector, divided into kb and ks.

(18) $$\left\{\begin{array}{c} \varepsilon_{x}\\ \varepsilon_{y}\\ \lambda_{xy}\\ \lambda_{yz}\\ \lambda_{xz} \end{array}\right\}=\left[\begin{array}{cc} \begin{array}{ccc} z & 0 & 0\\ 0 & z & 0\\ 0 & 0 & z \end{array} & \begin{array}{cc} 0 & 0\\ 0 & 0\\ 0 & 0 \end{array}\\ \begin{array}{ccc} \begin{array}{c} 0\\ 0 \end{array} & \begin{array}{c} 0\\ 0 \end{array} & \begin{array}{c} 0\\ 0 \end{array} \end{array} & \begin{array}{cc} 1 & 0\\ 0 & 1 \end{array} \end{array}\right]\left\{\begin{array}{c} \theta_{yx}\\ -\theta_{xy}\\ \theta_{yy}-\theta_{xx}\\ w_{y}-\theta_{x}\\ w_{x}+\theta_{y} \end{array}\right\}$$

Vaz [201] points out that now that the curvatures associated with the deformations $\gamma_{yz}$ and $\gamma_{xz}$ are only null if presented in Equation (19). The author cites that when it is valid, by the Kirchhoff hypothesis the rotations θ are given by the derivatives of *w*. As the γ distortions are not necessarily zero in the Mindlin theory, shear stresses and shear stresses will also not be zero.

(19) $$\{\begin{array}{c} \gamma_{yz}=w_{y}-\theta_{x}=0\\ \gamma_{xz}=w_{x}-\theta_{y}=0 \end{array}$$

Soriano [75] and Vaz [201] cite that through constitutive law, it is possible to obtain a general equation for the element (Equations (20) and (21)).

(20) $$\left\{\begin{array}{c} M_{x}\\ M_{y}\\ M_{xy} \end{array}\right\}=D\left[\begin{array}{ccc} 1 & v & 0\\ v & 1 & 0\\ 0 & 0 & \frac{\left(1-v\right)}{2} \end{array}\right]\left\{\begin{array}{c} \begin{array}{c} -\theta_{yx}\\ \theta_{xy} \end{array}\\ \theta_{xx}-\theta_{yy} \end{array}\right\}$$

where

(21) $$D=\frac{Et^{2}}{12\left(1-v^{2}\right)}$$

Equation (22) presents the summarized form of the equation, where {*M*} is the momentum vector at a point on the mean surface of the plate, [*E*] is the plate bending stiffness matrix by Kirchhoff theory and {$\varepsilon_{F}$} is the bending deformation vector of thin plates [75].

(22) $$\left\{M\right\}=\left[E\right]\left\{\varepsilon_{F}\right\}$$

For an isotropic element given by Equation (23), where K is the shear factor and G is the transverse modulus of elasticity, β is the shear deformation and KA represents a reduced area. In Equation (24), a new summarized equation for Mindlin theory is shown, where $\varepsilon_{C}$ represents the general shear stress rotation or deformation K [75].

(23) $$\tau =\frac{Q}{KA}=G\beta$$

(24) $$\left\{\begin{array}{c} Q_{x}\\ Q_{y} \end{array}\right\}=GKt\left\{\begin{array}{c} \beta_{y}\\ -\beta_{x} \end{array}\right\}=GKt\;\left\{\varepsilon_{C}\right\}$$

Considering the previous equations, it is possible to obtain the deformation energy for the element (Equation (25)) and the total potential energy (Equation (26)), where $\bar{q}$ is the transverse force per unit of a positive area in z-direction and $\tilde{I}$ is the identity matrix of the elastic coefficients in the general matrix [E] [75].

(25) $$U=\frac{1}{2}\int_{A}⎣-\theta_{yx}\;\theta_{xy}\;(\theta_{xx}-\theta_{yy})⎦\left\{\begin{array}{c} M_{x}\\ M_{y}\\ M_{xy} \end{array}\right\}dA+\frac{1}{2}\int_{A}⎣\beta_{y}-\beta_{x}⎦\;\left\{\begin{array}{c} Q_{x}\\ Q_{y} \end{array}\right\}\;dA$$

(26) $$\Pi =\frac{1}{2}\int_{A}⎣-\theta_{yx}\;\theta_{xy}\;\left(\theta_{xx}-\theta_{yy}\right)(w_{x}+\theta_{y})(w_{y}+\theta_{x})⎦\left[\begin{array}{cc} E & 0\\ \tilde{0} & G\tilde{K}t\tilde{I} \end{array}\right]\left\{\begin{array}{c} -\theta_{yx}\\ \theta_{xy}\\ \theta_{xx}-\theta_{yy}\\ w_{x}+\theta_{y}\\ w_{y}-\theta_{x} \end{array}\right\}dA-\int_{A}\bar{q}w\;dA$$

Soriano [75], Saliba et al. [212] and Vaz [201] mentioned that unlike Kirchhoff’s theory, the rotations θx and θy are independent of the displacement w (x, y). This independence allows us to formulate C0-continuity elements.

**Figure 21:**
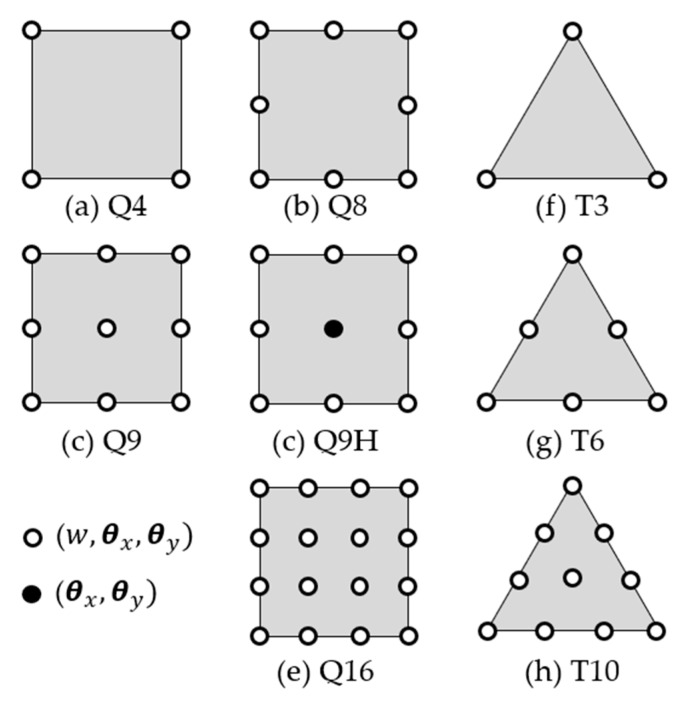
Finite elements based on the Reissner–Mindlin theory.

Through Reissner–Mindlin theory, several elements have been developed, which can either be curved or not. In Figure 21 some of the isoparametric elements applied to the plate theory are presented—independent approximations for w (x, y), θx (x, y) and θy (x, y), are easily written due to general parametric FEM formulation [75,212,214]. For elements Q4, Q8, Q9, Q16, T3, T6 and T10, all nodes have three degrees of freedom: one vertical displacement and two rotations. However, the element Q9H, called heterosis, has the peripheral nodes with three degrees of freedom and the central node with only two rotations 

Soriano [75] mentioned that for some reason, low order elements are subjected to locking or convergence ratio reduction. In order to identify the susceptibility to locking and the quality relation between the elements, the heuristic beam restraint ratio is generalized. For this, two constraints of shear stress are associated with each point of stiffness matrix integration; one related to $\beta_{y}$ and another to $\beta_{x}$. However, support constraints on the spatial distribution are not considered, these being dependent on the geometric distortion of the element; i.e., this distortion influences the ability of the element to represent constant or zero shear strain deformations.

#### 5.1.3. Theory of Kirchhoff versus Theory of Mindlin

Kirchhoff theory is suitable for a thin plate; Reissner–Mindlin plates; and thin and thick plates (also called semi-thick), but its application to thin plates requires special attention in order to make the elements capable of representing real-life cases [75,196,206,207,212,221]. Schneider, Kienzler and Böhm [213] cited that well-established standard theories for (linear geometry) homogeneous isotropic plate bending problems are: Kirchhoff theory, for neglecting the influence of shear deformations only suitable for very thin plates, and Reissner–Mindlin theory, which considers the influence of shear deformations and is used for thick plates. 

However, there are more factors to be considered, such as static or dynamic behavior, a plate made from a single material and layers coming from distinct materials (sandwich or laminated). Shear strain consideration is the most important issue in a dynamic behavior and/or in a sandwich plate [75].

### 5.2. Shell Element 

A shell is a two-dimensional planar solid whose thickness (h or t), usually measured along the z-axis, is much smaller than its length and width, both located in the *xy* plane [21,22,75,198,203]. This element is curved and can withstand bending and membrane effects, consisting of an average surface deformation for the element located on the same surface [21,75,201,222,223]. Examples of shell structures include acoustic shells, stadiums, large-span rooves, cooling towers, piping systems, pressure vessels, aircraft fuselages, rockets, water tanks, arch dams and many more. Even in the field of biomechanics, shell elements are used for the analyses of the skull, crustaceans’ shapes, red blood cells, etc. [75,109,199,200,224,225].

The shell element has probably generated more academic work in FEM technology than any other topic; however, it shows more computational barriers among all continuous structural elements, due to its curved geometry and the larger number of parameters involved [75,199,200,224]. 

**Figure 22:**
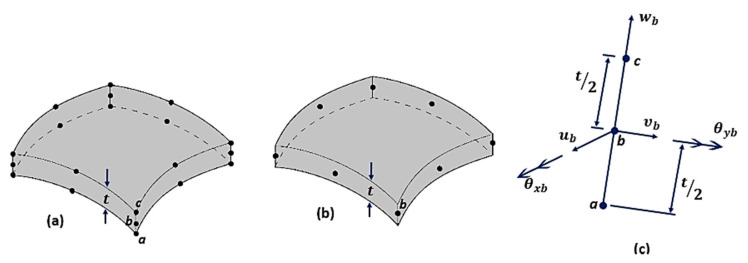
(**a**) 20-node isoparametric solid element; (**b**) reduction to eight nodes using a shell element; and (**c**) b-node representation.

According to Barbero [77], most of the composite structures are modelled using plate and shell elements. According to the author, this happens because, beyond reducing the numbers of nodes and elements, when compared to the solid element, it makes the modelling of thick laminates easy (Figure 22).

Its shape allows certain membrane tensile systems to act parallel to its tangential plane and become primary deformation carriers. In fact, the analysis of many fine elements is based solely on shell membrane theory, neglecting their flexural stiffness [22].

Mathematically, the shell element model is similar to the plate element, since it is common to consider null the transverse normal stress component [21,22,75,198]. The shell geometry can be defined by its average surfaces or just one of its outer surfaces, called the reference surface, along with the thickness of each point. In general, the average surface is used as the reference surface [75].

**Figure 23:**
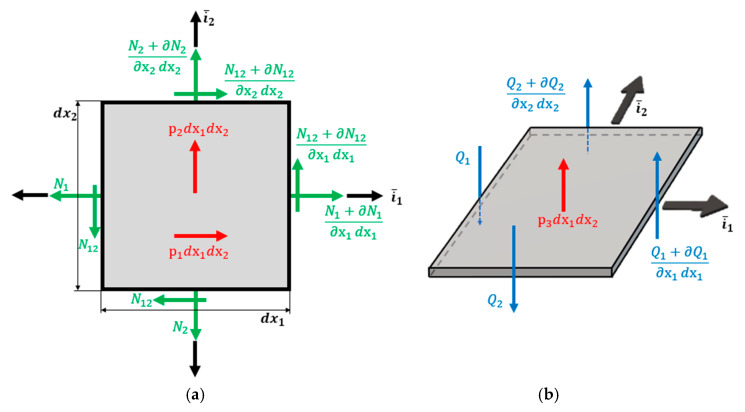
(**a**) Free body diagram for the equilibrium of in-plane forces and (**b**) free body diagram for the equilibrium of transverse shear forces.

According to Soriano [75], in the case of a shell element, bending is associated with the resultant loading forces (Mx, My, Mxy, Qx and Qy). In the case of small thicknesses, shell curvature radius expressions are identical to those from plate elements. Tensile components from a membrane effect, are the same as those occurring in the plane stress state, although they are considered by their results per unit length of the reference surface (Figure 23).

#### 5.2.1. Shell Theories

There are basically three coherent approaches for the shell element analysis. (a) Shell structure is faceted with flat elements, (b) via elements formulated on the basis of curved-shell theory or (c) degenerated three-dimensional elements [221,226,227]. 

##### The Theory of Flat Plate

The general theory of thin shell or flat shell was presented by H. Aron in 1874 and by A.E. Love in 1888, but it was only applied to solve engineering problems a century later. Similarly, to plate theory, plane shell theory basically differs from the idea of shear stress deformations, because in this case the analysis of shells with these elements is performed by superposing the membrane stiffness due to the plate elements [75,228]. Depending on the type of problem analyzed, the solutions obtained may depend on the discretization degree. 

In spite of the presented difficulties (discontinuity in the momentum of interface elements) these elements are applied in linear and nonlinear shell analyses [226]. Plane shell theory can be divided into sub-theories under the assumptions of Reissner–Mindlin (first order theories), higher order theories and discrete layer theories [75,222,229].

In this theory, shear stress deformations are neglected, assuming that a line segment perpendicular to the average surface of the shell remains straight and normal to this surface after its deformation. Differently from the Reissner–Mindlin hypothesis, which assumes that the segment remains straight, not normal to the middle surface though [75,229].

In higher-order theory, nonlinear polynomial laws are adopted to define the segment bending after deformation; physical models, though, are better represented in thematic models than in other theories. Discrete layers theory is suitable for laminated shells, with a linear displacement field adopted by segments and thickness imposing appropriate contact conditions at the ply interfaces [75].

##### Three-Dimensional Elements

When a shell analysis is done with three-dimensional finite elements, many numerical difficulties may occur due to the discretization along with thickness, leading to an equation system with a greater number of unknowns compared to the degenerate model using a reference surface [75,226].

All shell models have uncertainties when compared to three-dimensional elasticity theory; nonetheless, they have the advantage of operating over very small magnitudes, allowing easy calculation of stresses [75].

##### Degenerate Shell Element of the Three-Dimensional Element

In the three-dimensional, degenerated shell element approach, simply known as the degenerated shell element, the element behavior, towards independent displacements and rotations, is degenerated from three-dimensional tensions and deformations [226]. 

These elements have the advantage of requiring only a C0-continuous function, once equilibrium equations are second-order differential equations. The degenerative concept of finite element formulation was extended by several authors into a linear and nonlinear analysis of anisotropic laminated composite structures [226].

Modelling using the degenerated shell is adopted because it reduces computational time, referring specifically to data provision and analysis, as well for numerical reasons [75,226]. 

#### 5.2.2. Shell Element Types

**Figure 24:**
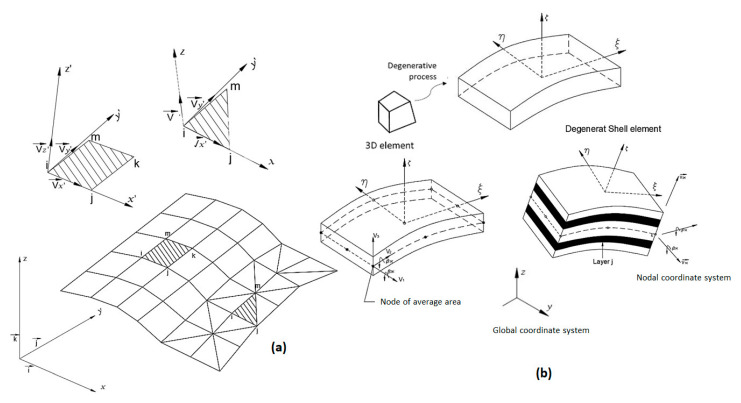
(**a**) Association of elements and (**b**) degenerate shell.

The classical shell formulation requires displacements of a fifth-degree polynomial; consequently, a high number of nodal parameters are needed for both thin and shallow shells. The most practical solution is to develop shell elements starting with associations of plane stress elements and plate flexion, or by using the degenerated, three-dimensional curved element on a surface, adopting kinematic and mechanical constraints (Figure 24), resulting in in-plane elements, curved elements (with Reissner–Mindlin hypotheses) and an axisymmetric shell with an asymmetric loading [75,221,222].

##### Flat Elements

According to Soriano [75], it is possible to combine plate elements with flat plane stress elements; thus, it is worked out with the plane stress resultants, considered plane elements. The plane element generates an approximation of the geometry in the curved shell discretization, replacing it by a set of facet elements, aside from displacement field approximations, inherent in finite element analysis.

**Figure 25:**
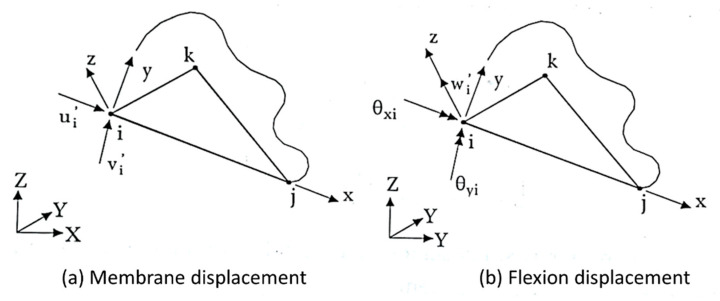
Triangle element in xy plane. Reproduced with permission [75].

This type of discretization requires a large number of elements with a refined polyhedral surface approaching the original mean surface. In this case, the triangular elements (Figure 25) better represent a double-curved shell geometry than the quadrilateral elements, which in this case are more interesting for single-curved shells and flat-shell discretization [75].

Considering many contributions from various elements, it is possible to determine a global stiffness matrix (Equation (27)), with [λ] representing a three-dimensional rotation matrix [75].

(27) $${\left\{u^{\prime }\right\}}_{i}={\left[r\right]}^{e}{\left\{u\right\}}_{i}={\left[\begin{array}{cc} \lambda & 0\\ \tilde{0} & \tilde{\lambda } \end{array}\right]}^{e}\;{\left\{u\right\}}_{i}$$

##### Curved elements (with Reissner–Mindlin Hypotheses)

According to Soriano [75], in 1968, Ahmad, Irons and Zienkiewicz developed a degenerated shell element, starting from a three-dimensional curved element. This degeneration became widely used with the knowledge of reduced/selective integration and evolved with complete integration of the mixed formulation, aiming to obtain robust elements. 

**Figure 26:**
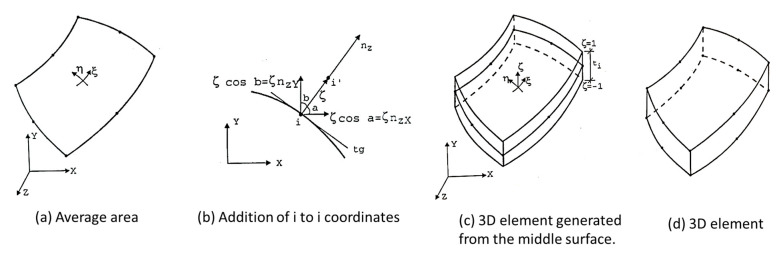
Curved element. Reproduced with permission [75].

Cook [21] cited that for curved elements, one can start from the middle surface, with the geometric definition and displacement field, adopting Reissner–Mindlin hypotheses, instead of three-dimensional explicitly degenerated element on its average surface by imposing a normal condition which maintains it straight, but not necessarily normal to this surface according to Reissner–Mindlin theory (Figure 26). In general, any other reference surface which is not necessarily the average one may be used as an outer surface. However, the average surface is usually adopted in the case of single-layer shells with homogeneous thickness [75].

According to Soriano [75] the main advantages of using the degenerate shell are:

➢ Working with the shell hypothesis from the beginning, obtaining, in a simple way, a wide range of elements;
➢ Developing curved elements that only need C^0^ continuity;
➢ Using only linear displacements and rotations as degrees of freedom, making it possible the use shell elements to discretize beam and plate elements;
➢ Considering the effect of shear strain on a wide variety of thicknesses.

Equation (28) shows the parametric form for a three-dimensional geometry, starting from the mean surface of a curved element, where ζ is the dimensionless coordinate of the z-axis with values at the outer surfaces of ±1; $t_{i}$ is the thickness at the nodal point *i*; $n_{zXi},\;n_{zYi},\;n_{zZi}$ are the z-axis directional cosines also at point *i*, components of the vector ${\left\{n_{z}\right\}}_{i}$.

(28) $$\left\{\begin{array}{c} X\\ Y\\ Z \end{array}\right\}=\overset{p}{\underset{i=1}{\sum }}N_{i}\;{\left\{\begin{array}{c} X\\ Y\\ Z \end{array}\right\}}_{i}+\zeta \overset{p}{\underset{i=1}{\sum }}N_{i}\;\frac{t_{i}}{2}\;{\left\{\begin{array}{c} n_{zX}\\ n_{zY}\\ n_{zZ} \end{array}\right\}}_{i}$$

Assigning the displacements according to the local axes x, y and z for u’, v’and w’ respectively, there are deformation vectors in a global reference and deformation components, respectively [75]. Based on these considerations and through generalized Hooke’s law, (assuming $\sigma_{z}=0$), one can obtain the general equation for a degraded shell (Equation (29)).

(29) $$\left\{\begin{array}{c} \sigma_{x}\\ \sigma_{y}\\ \sigma_{z}\\ \tau_{\mathrm{xy}}\\ \tau_{\mathrm{xz}}\\ \tau_{\mathrm{yz}} \end{array}\right\}=\frac{E}{1-\upsilon^{2}}\left[\begin{array}{cccccc} 1 & v & 0 & 0 & 0 & 0\\ 0 & 1 & 0 & 0 & 0 & 0\\ 0 & 0 & 0 & 0 & 0 & 0\\ 0 & 0 & 0 & \frac{\left(1-v\right)}{2} & 0 & 0\\ 0 & 0 & 0 & 0 & \frac{\left(1-v\right)K}{2} & 0\\ 0 & 0 & 0 & 0 & 0 & \frac{\left(1-v\right)K}{2} \end{array}\right]\left\{\begin{array}{c} \begin{array}{c} \begin{array}{c} \varepsilon_{x}\\ \varepsilon_{y} \end{array}\\ \varepsilon_{z} \end{array}\\ \gamma_{xy}\\ \gamma_{xz}\\ \gamma_{yz} \end{array}\right\}-\frac{E\alpha T}{1-\upsilon }\left\{\begin{array}{c} \begin{array}{c} 1\\ 1\\ 0 \end{array}\\ 0\\ 0\\ 0 \end{array}\right\}$$

From the curved shell element formulation with Mindilin theory for plates, Soriano [75] highlights the following differences:

➢ For plates, initially, shear rotations were separated from the plate and worked on the tension-deformation relationships with the resultant stress, excluding, consequently, integration along with thickness in the rigidity matrix and nodal forces equivalence expressions;
➢ For shells, working with total rotation and stress components results in expressions of rigidity matrix and equivalent nodal forces that require integration along with thickness. Note that plate elements could also be formulated the same way.

##### Asymmetric Shell with Asymmetric Loading

The use of structural axisymmetric (geometry and support conditions) and asymmetric loading leads to a simpler discrete model than the corresponding three-dimensional one. This simplicity is linked to a specific geometry and a smaller number of variables to be determined [75,230].

In 1963, Grafton and Strome presented the axisymmetric shell discretization, with asymmetric loading, in truncated cones corresponding to rectilinear finite elements according to a meridian shell, which has two nodal points and three displacements per node. Jones and Stone in 1966 modified the work from Grafton and Strome, considering curved elements according to the meridian; both authors considered the thin shell theory though [75].

**Figure 27:**
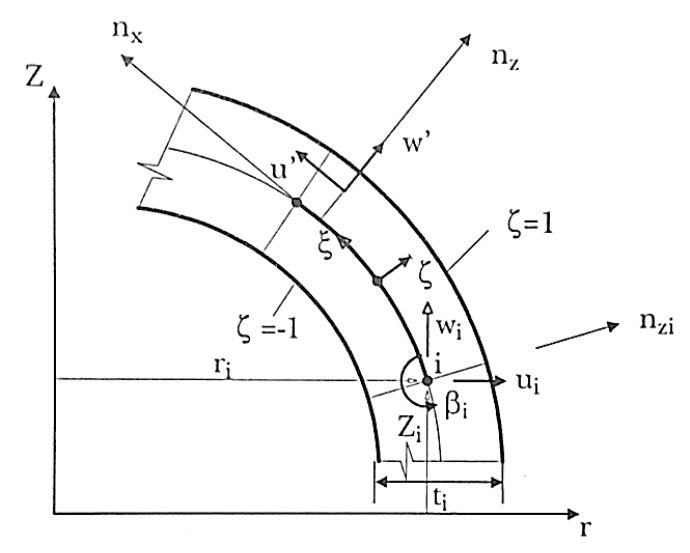
Asymmetric shell element. Reproduced with permission [75].

These elements of revolution (conical and curved elements) have nodal circles and not nodal points, as for plate elements, and in general, there are two nodal circles per element, which has two translations (radial and axial) and one rotation [21]. Figure 27 represents the axisymmetric shell element, where x and z axes are respectively tangent and normal to a meridian of a mean surface at each point r, θ and Z with radial direction and Z to axial [75].

The equation that defines the displacements’ interpolation is represented in Equation (30), where *u* is the radial displacement, w is the axial displacement and $\beta_{i}$ is the rotation of the nodal point i, according to the circumferential direction [75].

(30) $$\left\{\begin{array}{c} u\\ w \end{array}\right\}=\sum_{i=1}^{p}N_{i}^{\prime }\;{\left\{\begin{array}{c} u\\ w \end{array}\right\}}_{i}+\zeta \sum_{i=1}^{p_{2}}N_{i}^{\prime \prime }\;\frac{t_{i}}{2}{\left\{\begin{array}{c} n_{xr}\\ n_{xZ} \end{array}\right\}}_{i}\;\beta_{i}$$

Through all these considerations regarding stress and strain, the local and global referential are obtained by exclusion of $\gamma_{xy}\;\mathrm{and}\;\gamma_{yz}$, and by the exchange of y with θ, resulting in the axisymmetric shell general equation (Equation (31)) [75].

(31) $$\left\{\begin{array}{c} \sigma_{\theta }\\ \sigma_{x}\\ \sigma_{z}\\ \tau_{\mathrm{xz}} \end{array}\right\}=\frac{E}{1-\upsilon^{2}}\left[\begin{array}{cccc} 1 & \upsilon & 0 & 0\\ \upsilon & 1 & 0 & 0\\ 0 & 0 & 0 & 0\\ 0 & 0 & 0 & \frac{\left(1-v\right)K}{2} \end{array}\right]\left\{\begin{array}{c} \begin{array}{c} \begin{array}{c} \varepsilon_{\theta }\\ \varepsilon_{x} \end{array}\\ \varepsilon_{z} \end{array}\\ \gamma_{xz} \end{array}\right\}-\frac{E\alpha T}{1-\upsilon }\left\{\begin{array}{c} \begin{array}{c} 1\\ 1\\ 0 \end{array}\\ 0 \end{array}\right\}$$

### 5.3. Cohesive Elements

Cohesive elements, also called decohesion elements or interface elements, are useful in modelling adhesives, bonded interfaces, delamination and rock fracturing [58,65,231,232,233,234,235]. The constitutive response of these elements depends on the specific application and is based on certain assumptions about the stress and strain states that are appropriate for each application area. The nature of the mechanical constitutive response can be broadly classified based on [232,236,237]:

➢ Continuum-based modeling;
➢ Laterally unconstrained adhesive patche;
➢ Traction-separation based modeling.

**Figure 28:**
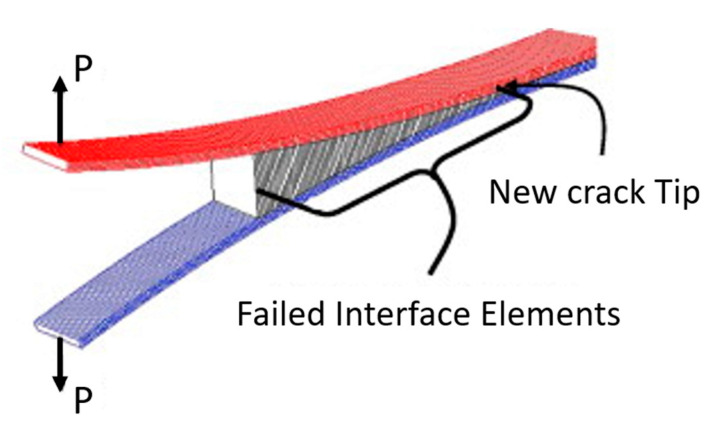
Interface element analysis of delamination in a double cantilever beam. Reproduced with permission [64].

In these approaches, cohesive elements are used to represent the behavior of a fracture, while traditional volumetric elements represent deformations of the continuous medium. Cohesive elements are inserted at the interfaces between pairs of adjacent volumetric elements in the finite element mesh (Figure 28) [238].

#### 5.3.1. Continuum-Based Modeling

**Figure 29:**
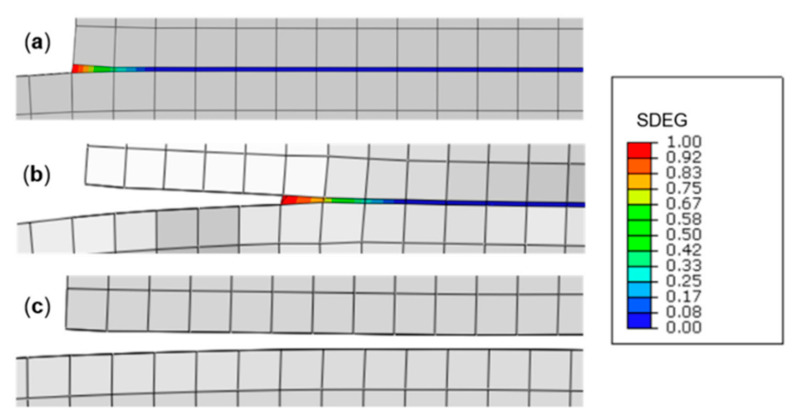
Progressive failure process of the adhesive layer in the lap-shear joint, (**a**) damage initiation at the overlap edges (SDEG-% = 0), (**b**) propagation towards the joint centre (SDEG-% ≈ 40), (**c**) joint failure (SDEG-% = 100). Reproduced with Creative Common License [239].

According to Joshi, Pal and Chakraborty [237], continuum-based modeling is used when the cohesive zone has a finite thickness such as a joining of two surfaces with the help of adhesive material such as glue (Figure 29). The thickness, stiffness and strength of a cohesive zone can be estimated using experimental methods. In the case of continuum modelling, one directs stress in the direction of thickness, and two shear stresses mutually perpendicular and along the plane of the adhesive are present.

The cohesive elements model the initial loading, the initiation of damage and the propagation of damage leading to eventual failure in the material [232].

**Figure 30:**
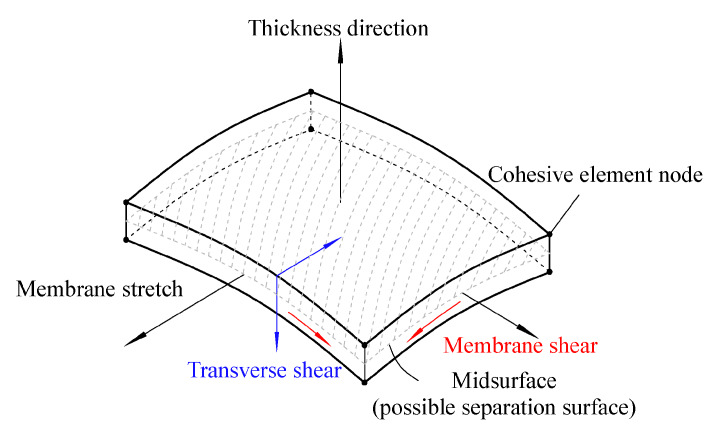
Spatial representation of CH3D8 (eight-node three-dimensional) cohesive element. Reproduced with Creative Common License [240].

The continuum-based can be applied in 2D and 3D problems. In 2D problems the continuum-based constitutive model assumes one direct strain (through-thickness), one transverse shear strain and all stress components to be active at a material point. In 3D problems it assumes one direct strain (through-thickness), two transverse shear strains, and all stress components to be active at a material point (Figure 30) [232,237].

#### 5.3.2. Laterally Unconstrained Adhesive Patche

**Figure 31:**
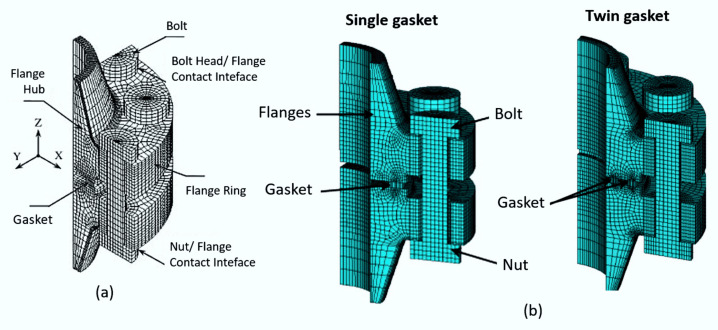
Typical application involving gaskets, (**a**) Reproduced with permission [241] and (**b**) Reproduced with permission [242].

This approach is appropriate for modelling joints with gaskets (Figure 31). The macroscopic properties of the gasket, such as strength and stiffness, are used for the analysis. Only unidirectional stress along the through-thickness direction is considered in the analysis. The nonlinear and hyperelastic behavior of the materials used for gaskets—rubber, foam, etc.—can be captured in the constitutive relations used for the modelling techniques for laterally unconstrained adhesive patches [237]. The constitutive responses of gaskets modelled with cohesive elements can be defined using only macroscopic properties such as stiffness and strength [232]. 

#### 5.3.3. Traction-Separation-Based Modeling

**Figure 32:**
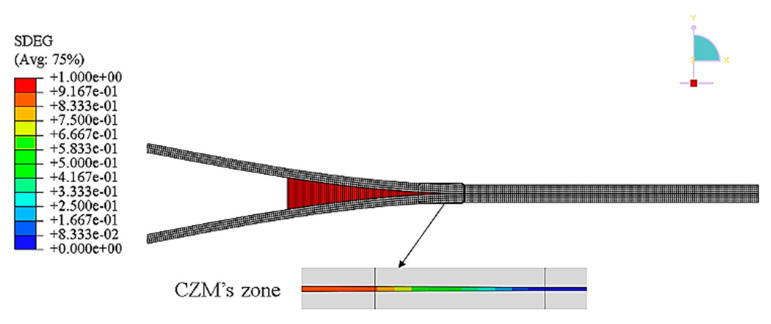
Damage of cohesive elements in the wake of delamination front. Reproduced with permission [246].

The modelling of bonded interfaces in composite materials often involves situations wherein the intermediate glue material is very thin and for all practical purposes may be considered to be of zero thickness. Therefore when the macroscopic properties of the material, such as the rigidity and strength of the adhesive material, are not important, traction-separation-based modelling can be used (Figure 32) [60,63,232,237,243,244].

In cases of the macroscopic material properties are not relevant directly, the analysis should be based on concepts derived from fracture mechanics—such as the amount of energy required to create new surfaces [232,245].

The cohesive elements model (CZM) models the initial loading, the initiation of damage and the propagation of damage leading to eventual failure at the bonded interface. The behavior of the interface prior to initiation of damage is often described as linearly elastic in terms of a penalty stiffness that degrades under tensile and/or shear loading but is unaffected by pure compression [232,247].

According to Abena, Soo and Essa [60], the limitation of this approach is the inability to represent the beginning of the damage and the propagation of the failure under compression and the inability to produce any stress related to a membrane response. In contrast, elements representing the surrounding phases (matrix and fibre) are able to fail under compression and a membrane response, and are consequently deleted during the analysis. Therefore, the cohesive elements could remain in the model even if their surrounding elements fail. When this happens, the cohesive elements lose their aim, since they are not linking matrix and fibre any more, and they also usually experience excessive distortion since their nodes become free to move [60].

During modelling of the joints under traction-separation technique, before damage initiation, linear elastic behavior is assumed [60]. In 2D problems, the traction-separation-based model assumes two components of separation (one normal to the interface and the other parallel to it), and the corresponding stress components are assumed to be active at a material point. In 3D problems there are three components of separation (one normal to the interface and two parallel to it), and the corresponding stress components are assumed to be active at a material point [60,232]. 

The linear elastic behavior before the initiation of damage can thus be governed by the constitutive relations, as given by Equation (32) [60], where $\sigma_{n}$ is the normal stress along the local direction 3 (through-thickness), and $\sigma_{s}$ and $\sigma_{t}$ are the shear stress components along the local directions 1 and 2, respectively. The $\varepsilon_{n}$ is the normal strain along the local direction 3, and $\varepsilon_{s}$ and $\varepsilon_{t}$ are the shear strain components along the local directions 1 and 2, respectively.

(32) $$\left\{\begin{array}{c} \sigma_{n}\\ \sigma_{s}\\ \sigma_{t} \end{array}\right\}=\left\{\begin{array}{ccc} D_{nn} & D_{ns} & D_{nt}\\ D_{sn} & D_{ss} & D_{st}\\ D_{tn} & D_{ts} & D_{tt} \end{array}\right\}\;\left\{\begin{array}{c} \varepsilon_{n}\\ \varepsilon_{s}\\ \varepsilon_{t} \end{array}\right\}$$

**Figure 33:**
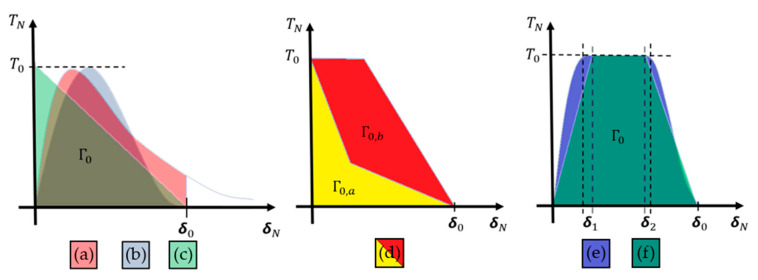
Typical traction separation laws according to: (**a**) Needleman 1987, (**b**) Needleman 1990, (**c**) Hillerborg 1976, (**d**) Bazant 2002, (**e**) Scheider and Brocks 2003, (**f**) Tvergaard and Hutchinson 1992.

The failure mechanism consists of damage initiation criterion and damage evolution law. The damage initiation can be governed by the criteria of maximum stress and maximum strain [60]. Regarding damage evolution law, different cohesive laws have been proposed in the literature, but normally, assumptions of zero adhesive thickness are made (Figure 33) [234,248,249]. 

Once the damage criteria are met in any one of the modes, then the stiffness starts degrading, causing a gradual failure. This is also called as softening [237,250]. 

According to Arafah [248], Schwalbe, Scheider and Cornec [251] and Budiman et al. [252], the traction separation law (TSL) can be described by the following parameters: the critical separation ($\delta_{0}$)—that is, the maximum displacement jump across the crack at which the cohesive element becomes completely broken; the cohesive strength ($T_{0}$), which is the maximum traction at the crack plane; and the cohesive energy $\Gamma_{0}$, which is the amount of energy consumed to create new crack surfaces (i.e., separation energy similar to Griffith’s fracture concept). The cohesive energy can be calculated from the area under the traction separation law T (δ), as in Equation (33).

(33) $$\Gamma_{0}=\int_{0}^{\delta_{0}}T\;\left(\delta \right).\;d\delta$$

**Figure 34:**
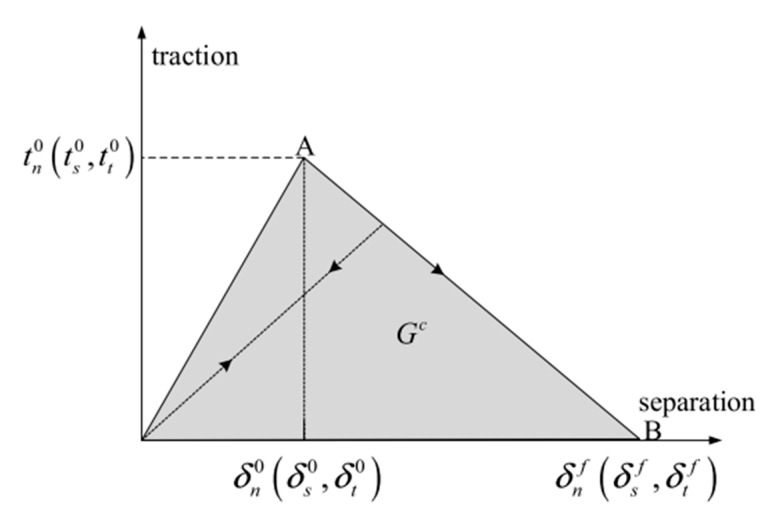
Typical traction–separation response. Reproduced with Creative Common License [249].

The initial response is assumed to be linear, and once a damage initiation criterion is met, damage can occur according to a user-defined damage evolution law (Figure 34) [249]. 

In the case of mixed mode loading, a tangential separation mode, usually designated Mode II and Mode III, accompanies the normally considered crack opening (Mode I) [233,251]. In linear elastic fracture mechanics, a phase angle $\left(\Psi_{\mathrm{LEFM}}\right)$ can be define by Equation (34) Figyre, where K_I_ and K_II_ denote the stress intensity factors for crack opening Modes I and II respectively [250].

(34) $$\Psi_{\mathrm{LEFM}}=\arctan\left[\frac{K_{II}}{K_{I}}\right]\;\;\;\;$$

In the context of the cohesive model, a tangential displacement $\left(\delta_{t}\right)$ represents the additional shear mode and is superimposed on the displacement normal to the crack plane $\left(\delta_{n}\right)$ (or plane of expected damage in the absence of a pre-existing crack) (Equation (35)) [250].

(35) $$\Psi =\arctan\left[\frac{\delta_{t}}{\delta_{n}}\right]$$

#### 5.3.4. Cohesive Element Types

**Figure 35:**
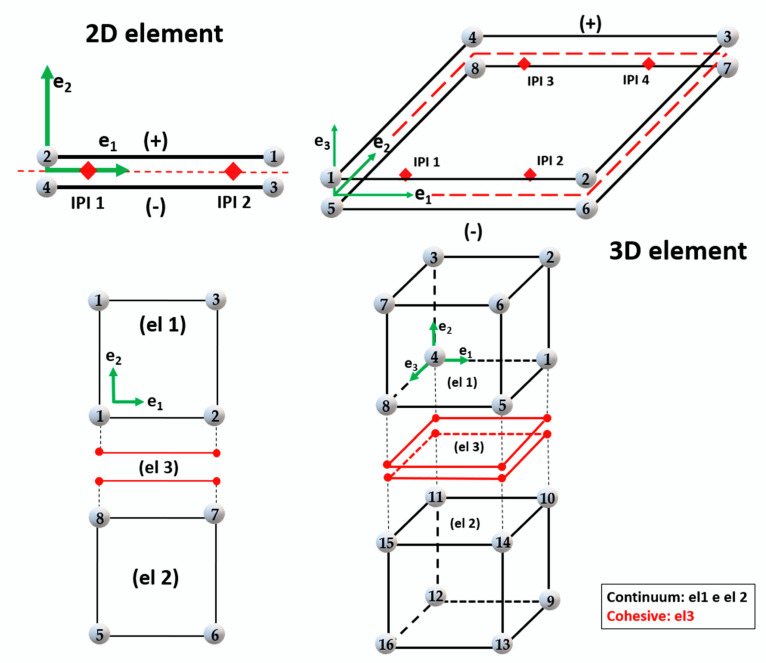
Cohesive element nodes and integration point positions, and connecting cohesive and continuum elements.

The cohesive elements can be defined as 2D or 3D elements. The 2D has four nodes and two integration points with a linear displacement formulation. The 3D can be constructed using eight nodes and four integration points with linear displacement formulation (Figure 35). The local coordinate system of the cohesive element could be defined with respect to the initial configuration or the actual configuration (i.e., moving coordinate system) (Figure 35) [240,248]. For the aim of calculating the stresses and separations of the cohesive element, they are connected to the adjacent continuum elements by sharing the respective common nodes (Figure 35) [248].

**Figure 36:**
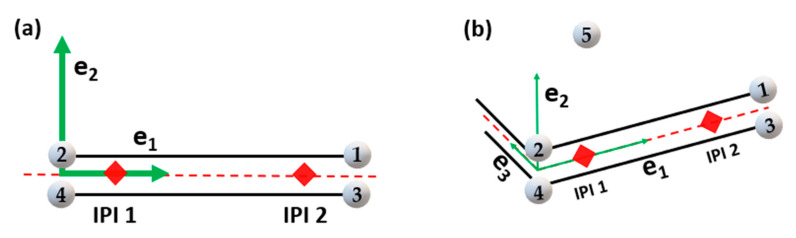
Cohesive element (**a**) plane stress/strain models and (**b**) shell models.

Modelling using the 2D cohesive elements has two options: the plane strain/stress and shell model. The difference between them is that the shell element is defined in the three-dimensional space. Therefore, any separation may be in-plane or out of the plane, and the in-plane direction must be defined by the user, which can be done by a fifth node, as shown in Figure 36 [250].

## 6. Main Applications of Finite Elements in the Study of Composite Materials

Every industrial sector feels over the years an increasing demand for innovative products that outperform competitors and meet market needs. For many design applications, resistant and lightweight materials are required, which makes laminates the ideal solution. However, new product developments and launches, and new technologies, cannot compromise product quality, reliability and speed [253].

The use of composites is growing rapidly in many industries, with new technologies becoming necessary for design, analysis and optimization. However, mechanical designers who work with steel and metals in general do not have many problems predicting the behaviors of these materials in use, because they are considered isotropic materials. Composite materials do not work the same way, once they are made from distinctive constituent materials [254]. Furthermore, their manufacturing is very expensive, making impractical the construction of real-size or scale-size prototypes.

In this context, numerical simulations evolved, mainly using the finite element method (FEM), and became a valuable tool to reduce project costs, enabling real-size components and equipment, boundary and entry conditions to be easily and quickly modelled. The usage of FEM to help engineering projects has increased over the past decades, due to computational capacity and data-processing performance, reduction project elaboration time and low costs.

Conventional software packages that use FEM in structure analysis, temperature, air and fluid fluxes, were not developed for composite materials, because of the fiber weaving and the microscale simulation approach needed. Acknowledging this deficiency, software providers started to include and add specific tools making composite analysis possible [254], owing to huge market demand for specialized tools to solve the constructive and operational particularities of these materials.

With this in mind, we can cite customized algorithms developed to work with well-known software such as Abaqus and HyperSizer, and add-on modules; for example, Ansys Composite Prep/Post, NISAII/COMPOSITE from Cranes Software Inc, FiberSIM from Siemens, Helius-Composite from Autodesk and GENOA from Altair and Laminate Tools from Anaglyph [255,256,257,258,259,260,261]. 

**Figure 37:**
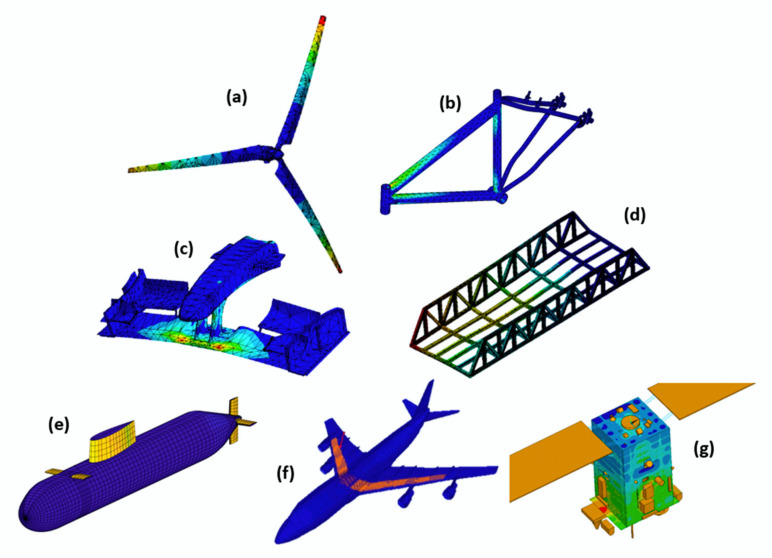
Some fields of FEM’s application to composites: (**a**) wind power, (**b**) sports, (**c**) automotive, (**d**) construction, (**e**) naval. Reproduced with Creative Common License [267], (**f**) aeronautics. Reproduced with permission [268] and (**g**) space. Reproduced with Creative Common License [269] industries.

As already mentioned, the field of application of composite materials is very wide (Figure 37); consequently, the FEM software for laminate analyses has to meet all these specificities [262,263,264,265,266]. 

### 6.1. Aronautical

As a way to demonstrate the importance of the use of FEM analysis in composite materials, there is the aeronautical sector, which could increase the percentage of laminates in aircraft—an advance impossible to be made with prototypes because of their high costs and very long manufacturing time.

**Figure 38:**
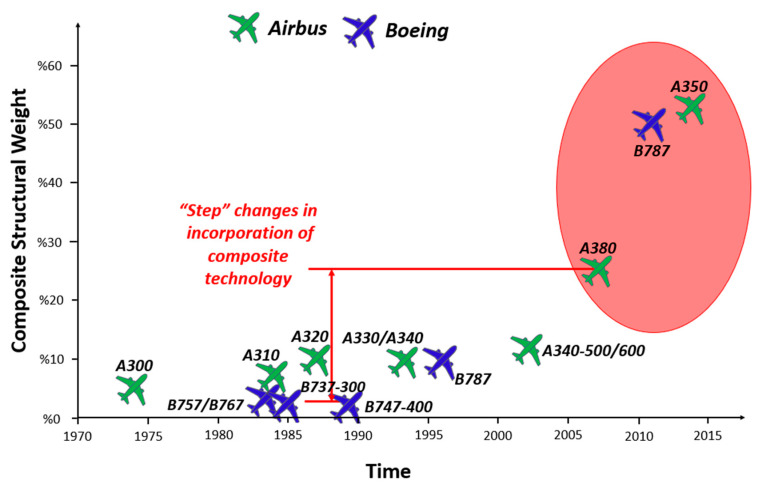
Use of composite materials in aircraft over the years.

After the 2000s, the aeronautical industry increased composites’ presence by structural weight by ≈40% [270] (Figure 38). This coincides with the increase in the processing capacity of computers and the evolution of numerical simulation software dedicated to composites.

According to Vijaykumar Rayavarapu [271], Hindustan Aeronautics Limited R&D—manager, a major advancement of simulation for this sector is the possibility to predict the best geometries and materials for withstanding bird impacts, which according to Zhou, Sun and Huang [272] is a recurring and problematic phenomenon in aviation worldwide, because a bird impact causes structural, serious damage, particularly threatening the safety of aircraft and passengers; e.g., loss of aircraft and even the loss of lives.

Rayavarapu said, “In the past, the only way to determine whether composite aircraft components could withstand bird strikes was with time-consuming physical tests. Now, engineers use simulation to get the design right the first time. Bird strike simulation saves the companies design time and thousands of dollars per test of composite helicopter/aircraft components.”

According to Bouvet, Rivallant and Barrau [273] and Sun et al. [249], another problem for companies that use composites in their products is the impact caused by the tools falling on the components during their assembly or maintenance (low velocity impact). Composite laminates have brittle behavior and can undergo significant damage in terms of matrix cracks, fiber breakages or delamination. This damage is particularly dangerous because it drastically reduces the structural integrity of the structure, and at the same time can leave very limited visible marks on the surface impacted [274,275]. 

Through the start-up Carbon, another great advancement was achieved by the use of FEM to reduce cargo weight to increase aircraft fuel efficiency. For them, “Lightweighting is one of the most important trends in the aerospace industry today, as jet manufacturers and their suppliers work to reduce the overall weight of planes and improve their fuel efficiency, include reducing the weight of the cargo” [276].

Huang [277] and Yang [278] still commented on the introduction of nanostructured materials in aircraft, which will help to increase the resistance and decrease their structural weight. These composites taken with the multifunctional ones have been the subjects of several studies in the last decade, as they present significant improvements in mechanical, chemical, electrical and thermal properties; in other words, not only mechanical support functions, but also integrated functions [278,279,280,281,282,283].

**Figure 39:**
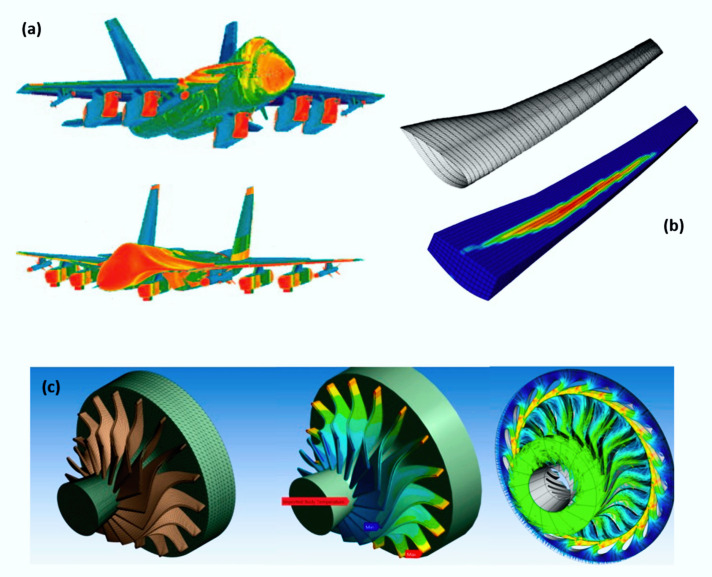
Some applications of FEM in composites in the aeronautical industry: (**a**) fluid–structure. Reproduced with Creative Common License [288], (**b**) wing geometry. Reproduced with permission [289] and (**c**) tube rotor blade improvement. Reproduced with Creative Common License [290].

In general, aeronautical companies use FEM in structural analyses, vibrations, dynamic buckling and fluid flows in the fuselage, wing, turbine and other parts (Figure 39) [284,285,286]. These analyses help determine the best geometry and material for each part of the aircraft, and understand how it will behave in service; for example, how the pressure and drag force will affect the aircraft during a flight [285,287].

### 6.2. Space

**Figure 40:**
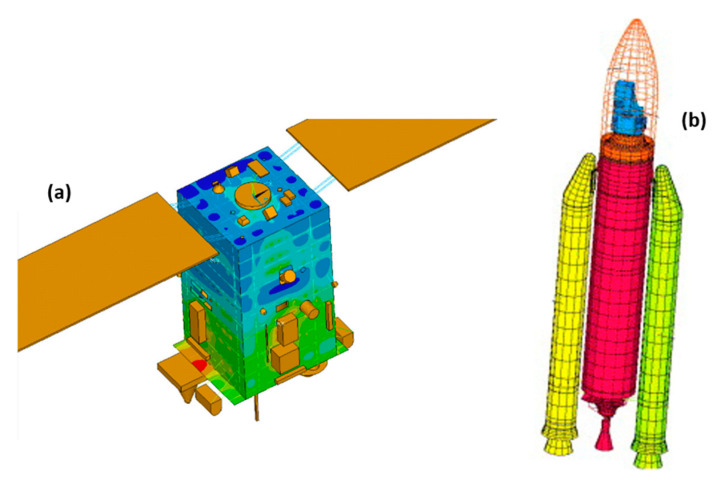
Some applications of FEM in composites in the space sector: (**a**) satellite. Reproduced with Creative Common License [269], and (**b**) coupled launcher–satellite structure. Reproduced with permission [295].

The space sector, and the aeronautical sector, have benefited greatly from the advances of numerical simulation, because nowadays they can simulate a structure or component of a vehicle and test what the best geometry or material for it will be. In addition, there is the possibility of simulating various types of environments, such as the vacuum of space, solar radiation and even conditions from other planets (Figure 40) [90,291,292,293]. According to John Thornton, chief engineer of Astrobotic Technology Inc. [294], “Using design and simulation tools is possible designed and refined a lightweight spacecraft able to withstand static acceleration and dynamic random vibration loads of launch while maintaining an acceptable level of safety.” He still complements simulations for helping to reduce costs related to prototypes and physical testing.

### 6.3. Automotive

Another sector that has been applying composites to its products is the automotive one; they use FEM for the bodies of the cars and engines, mainly for impact tests. According to Jung [296] they predict damage under impact loads on automotive components, as impacts can reduce the structural integrity of composite components. Impact damage induces a variety of failure modes: matrix cracking, fiber breakage, and delamination. Among these failure modes, delamination is the most significant because the interlaminar mechanical properties are much weaker than those the rest of the composite, and it is difficult to visually detect this damage from the outside [296,297,298].

One of the applications of FEM in this scenario is to understand the behavior of the material during an impact, as in the work developed by Chiacchiarelli, Cerrutti and Flores-Johnson [299], in which they used layers with the same density but different compressive strengths in the direction of load, in a multilayered, rigid polyurethane foam block subjected to impact, since, by its excellent energy absorption capacity, it can be used as a high-level coating resistant to collisions, which makes it attractive for the automotive sector.

**Figure 41:**
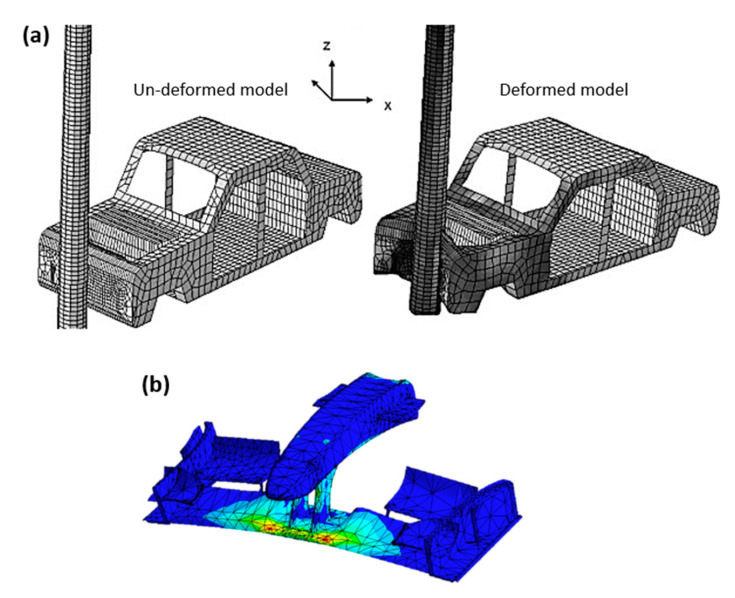
Some applications of FEM in composites in the automotive sector: (**a**) impact. Reproduced with Creative Common License [266], and (**b**) car racing.

In the area of super-sport and competition cars, such as those for Formula One racing, it is very advantageous to use simulations to find the best geometry of a car, since a few seconds can define the winner (Figure 41) [253,282,287,300,301,302].

According to Sandeep Sovani, director of Global Automotive Industry-ANSYS [253], “Simulation is key to solving issues upfront in the design phase. Companies that fine-tune auto body and chassis can reduce fuel consumption and build in reliability upfront in the design process with simulation.”

As examples the Sovani cite, “The Tier 1-supplier DENSO embeds CAE into all phases of its product development process, improving quality and reducing time to market along the way. Advanced virtual analysis enables such pacesetters to create category-changing; the KTM Technologies incorporated radical composites into a sports car, which called for new design, analysis and optimization technologies. Created using simulation, the product struck a fine balance between requirements, performance and costs while exceeding customers’ requirements; the ZF-TRW study the friction-induced brake squeal, for grow important as other vehicle noise sources are mitigated because of this the ZF-TRW engineers accurately simulated squeal and automated the simulation process while reducing time and money spent on validation testing. Performing simulation early in the design process helps to avoid costs associated with multiple prototypes, rework and tooling changes; and Valeo used nonlinear best practices to simulate thermoplastic snap- fits, leveraging HPC that shrunk simulation time by 50 percent” [253].

### 6.4. Naval

**Figure 42:**
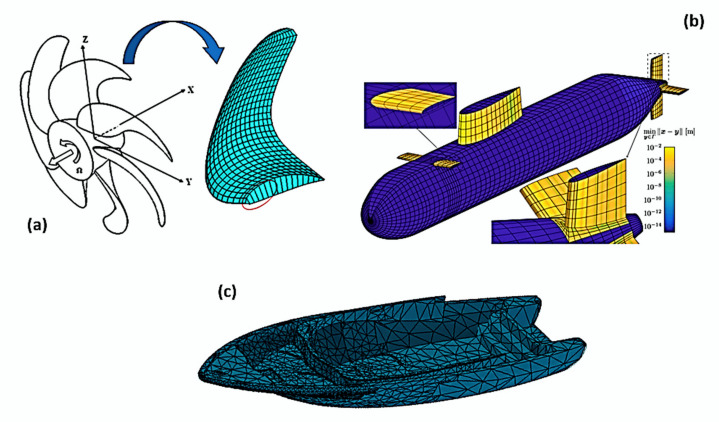
Some applications of FEM in composites in the naval sector: (**a**) propellers. Reproduced with permission [303], (**b**) submarines. Reproduced with Creative Common License [267] and (**c**) ships.

In the naval sector, the use of composites has been growing with the aid of FEM, with the main research being for marine propellers, ships and submarines (Figure 42). This research field uses FEM either to determine the mechanical properties or to predict the vibrations of its vessels, besides optimizing its structural weight [69,258,303,304,305,306,307].

### 6.5. Energy

**Figure 43:**
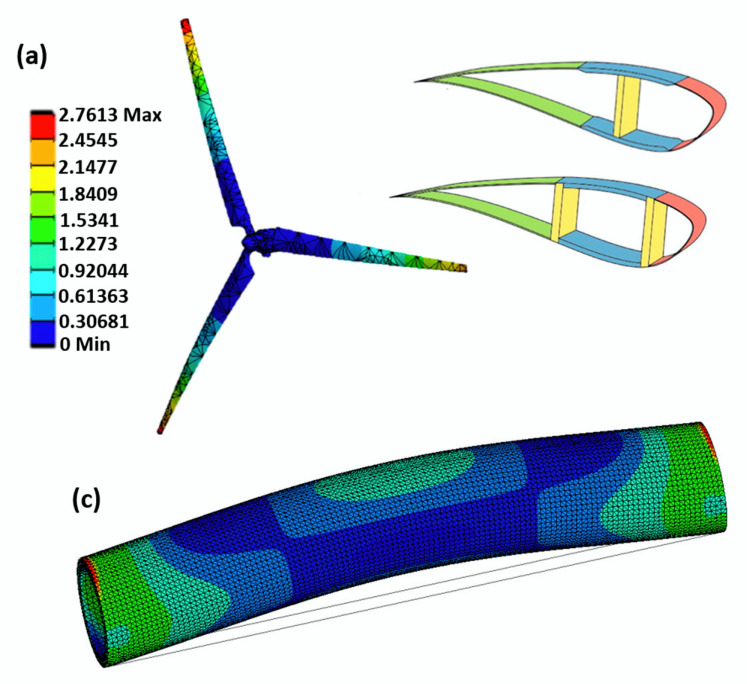
Some applications of FEM in composites for the energy sector: (**a**) wind turbines. Reproduced with permission [310] and (**b**) pipes for the oil and gas industry.

In the energy field, composites have been gaining space in the wind sector (Figure 43a), mainly due to the fabrication of the blade turbines, and with the help of FEM it is possible to predict how the wind; the heat; and the sun’s radiation, centrifugal force and gravity load will affect the blades—and the ideal geometry to optimize the turbine [46,308,309,310]. 

In addition, Oliveira [311] cited a major innovation of the WEG company, which “wanted to replace the steel cover that contains the rotor coil head with an alternative material in a new line of turbogenerators. The engineering team explored the use of a pre-impregnated composite material in the form of a banding tape instead of a retaining ring. The result was a fully validated component with a lower rotor mass that is also cost- effective to manufacture. New materials, like the composites in this case, can reduce feedstock costs by as much as 77 percent and wound rotor manufacturing costs by 18 to 20 percent.”

Another branch of the energy sector that has turned its attention to laminates is the oil and gas industry (Figure 43b), as they have been looking for deeper oil fields, and therefore, alternatives to the traditional armor wires are needed, due to a more severe environment. The traditional armor wires made from carbon steel are capable of operating at sea depths of 2.4 km but pose limitations, such as tensile fatigue, buckling and corrosion and excessive weight; thus, one option is the flexible hybrid composite pipe [312,313,314,315,316,317]. According to Anderson et al. [314] the combination of ultra-deepwater (>1500 m) applications and large pipe diameter requirements presents severe engineering challenges for rigid pipe technology. To address those needs, General Electric, with the support of a research partnership with Secure Energy for America, has a development program to generate a flexible pipe with an internal diameter of greater than seven inches for ultra-deepwater applications, and for this they use the FEM.

### 6.6. Civil Construction

**Figure 44:**
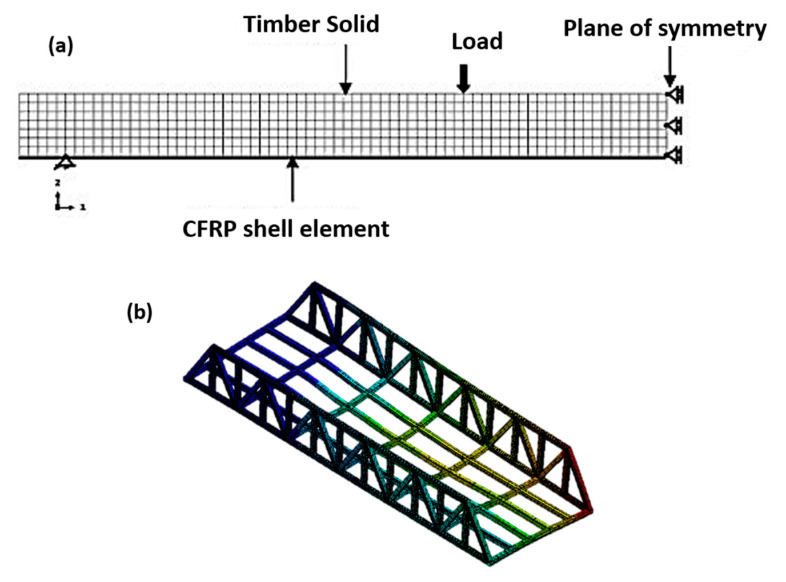
Some applications of FEM for composites in construction: (**a**) Strengthening flexural solid timber beams with CFRP. Reproduced with permission [323] and (**b**) bridges.

In civil construction, FEM has been used over the years for the structural calculations of buildings, bridges loads, etc., both of brickwork and wood (Figure 44). For designers, the advance of FEM in composites has helped to elaborate hybrid constructions, wherein fiber-reinforced polymeric composites are used as reinforcements, for example, of bridges and columns, besides helping analyze the effects of natural events such as earthquakes, tornados and hurricanes on builds [142,318,319,320,321]. In addition, as in the aerospace sector, the new nanostructured composites have been helping to reduce the structural weights of the constructions [277,322].

### 6.7. Sports

**Figure 45:**
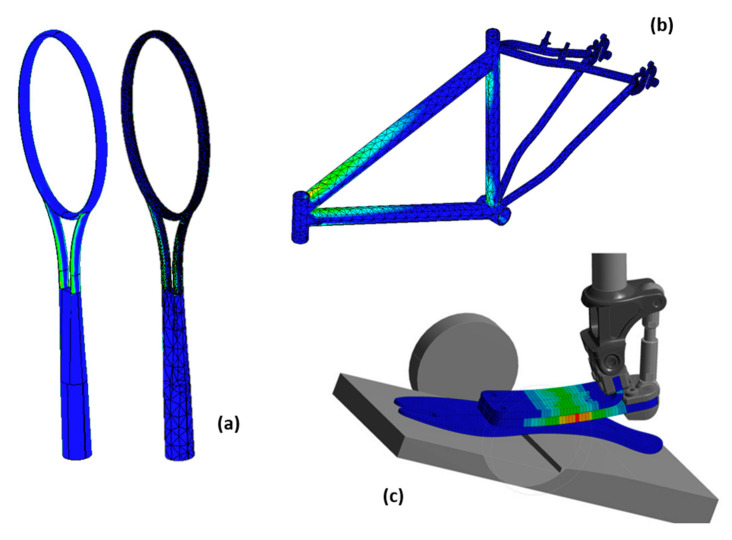
Some applications of FEM for composites in the sport sector: (**a**) a tennis racket, (**b**) a bicycle and (**c**) a prosthetic. Reproduced with Creative Common License [331].

Composite materials have not only been gaining a place in the large industrial sectors, but today they have been sports sector favorites as well, especially in the area of high performance or high-level sports. This field has been using composite materials to reduce equipment weight; take as examples, bicycles, tennis racquets, skis and even racing prosthetics (Figure 45). This field uses FEM in its projects as a way to determine geometries that have high resistance and lower weight to improve the athletes’ performances [253,324,325,326,327,328,329,330].

For KTM Technologies GmbH company, “Designers must predict how well the finished product will perform in the real world, such as on a race track or road. Predicting failure, delamination, ultimate strength and other development variables is critical before prototype and manufacturing stages” [253].

Scholz et al. [324] wrote: “Composites typically possess a superior strength to weight characteristic compared to monolithic materials and offer excellent biocompatibility. They are, therefore, favorable for both hard- and soft-tissue applications and the design of prostheses. In particular, the development of specifically designed carbon fiber sports prostheses now allows lower-limb amputees to actively participate in competitive sports. Sensory feedback systems, porous composite materials for tissue engineering and functional coatings for metallic implants are further developments anticipated to be introduced in next-generation orthopedic medicine.”

### 6.8. Manufacturing

**Figure 46:**
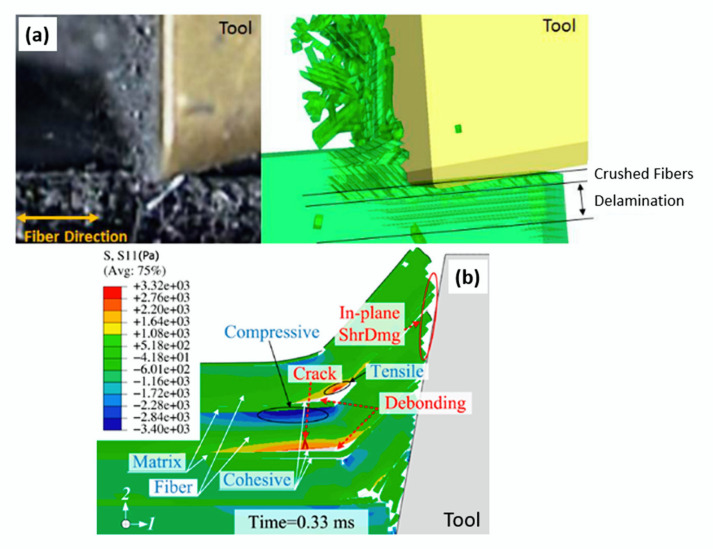
Example of damage cause by machining, (**a**) Reproduced with Creative Common License [333] and (**b**) Reproduced with permission [334].

Due to so many composite applications, the manufacturing sector has studied ways to minimize the damage generated during manufacturing processes, especially in machining (Figure 46). This concern is due to the damages that the process causes to laminates, such as delamination, interlaminar fissures, fiber–matrix displacement and thermal damage [332,333,334,335].

**Figure 47:**
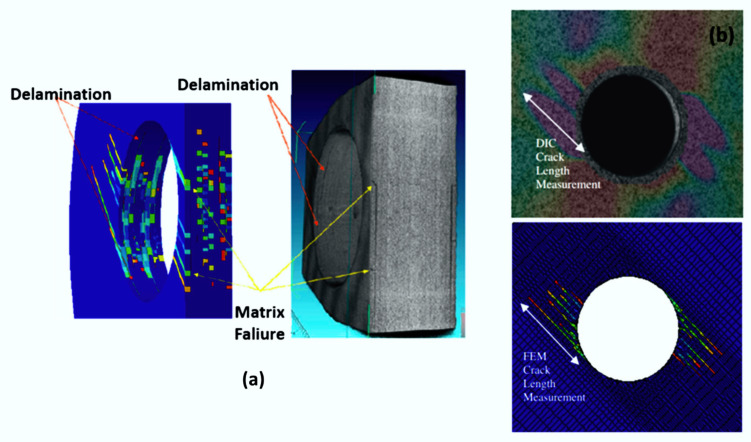
(**a**) Damage occurred in the FEM (left) and in the mechanical test (right). (**b**) Digital Image Correlation with overlapped shear strain (above) and FE damage (below) for the OHT coupon at 1,000,000 cycles. Reproduced with permission [338].

Another concern is the quality shown in the machined pieces; in this case, these pieces are perforated because the drilling process is the most used one for laminates, since most of the composite joints are bolted joints [332,336,337]. For this, static and dynamic mechanical tests are simulated in order to verify the damages caused by drilling and propagated by the test (Figure 47).

### 6.9. High-Performance Electronics

One of the most recent fields of composite application is high-performance electronics, because modern electronic devices rely on novel composite materials to achieve superior performance [339,340].

According to Sudhir Sharma, director of high-tech industry strategy and marketing of ANSYS, “Today, high-tech companies turn to advanced lightweight, yet strong, materials to create flexible mobile and wearable electronics. However, a range of complex issues must be considered when evaluating new materials—including electrical conduction properties, structural strength, dimensional stability over time and resistance to thermal build-up. The high-tech engineers simulate the electrical performance and thermal performance of electronic systems and devices. Design for manufacturability is also important” [313].

Sharma cited two examples of research using FEM in this area; the first case involved 3M, which published a groundbreaking study on how a novel embedded-capacitance composite material affected the electrical performance of a printed circuit board. The second is a study by the University of Pittsburgh and Carnegie Mellon University, wherein the engineers use FEM to assess the performances of new nanocomposites that have the potential to revolutionize power transformer technology.

Another research area that is growing in electronics regards polymer composites of high thermal conductivity, commonly used in many industries for renewable energy systems and electronic systems, such as solar cells, light-emitting diodes, Li-ion batteries and microelectronic packaging. This research is due to the potential applications as flexible polymers in electronic packaging and encapsulations, and satellite devices, because the local overheating and heat accumulation that occurs due to increasing power density may lead to the degradation and failure of functional systems [341,342,343]. 

## 7. Conclusions

In this article, the main contributions in the field of composite simulation were reviewed and presented, such as the specific characteristics, theories and constitutive relationships of the composite materials, and the types of elements and failure criteria used in each case. In addition, the main industrial sectors that have been using composite simulation as an important part of project development and study with these materials were also described.

At first, the definitions and characteristics that these materials can have were briefly commented on, as were the most usual configurations that are used in the various industrial sectors.

After that, the different types of models used in the simulations of composite materials, called micromechanics, homogeneous equivalent and the combination between them, were detailed. Starting with the definitions and concepts involved in micromechanical modeling, which individually approach the properties of each constituent material (fiber and matrix) and the interface between them, we presented a model with which it is possible to study local defects and failure mechanisms between the components; however, it demands high computational performance. Continuing with the definition of homogeneous equivalent modeling—it disregards the difference between constituents and treats the material as homogeneous, decreasing the demand for computational performance and making it impossible to study the effects locally.

After that, in the same section, the different levels of simulation that can be used in composite materials simulations were approached. Starting with simulations at the micro level, which focus on the interaction and interface between a fiber filament and the matrix, we then moved on to simulations at the meso level, approaching units of material volume and the relationships between the layers that make up the material; and finally, we described macro level simulations, which consider these materials as homogeneous.

Then, the constitutive relationships that can govern mechanical properties of these materials were discussed, presenting the differences between them one by one. We started with the constitutive relationship of an anisotropic material, followed that by an orthotropic material and finished with a transversely isotropic material.

In sequence, the article reviewed the various failure mechanisms that these materials can present, those being the failures in the fibers and in the matrix, due to loads that can be of compression, tension and/or shear. We also approached the various failure criteria proposed in research, such as Hill, Tsai–Hill, Tsai–Wu, Hashin-Rotem, Hashin, maximum stress, Hoffman, maximum stress, Hou, Puck–Schürmann, Chang-Chang, Linde, LaRC03, LaRC04, Maimi, Hart-Smith and Yeh-Stratton—each trying their best to get closer to the experimental results. Among all of those, maximum stress, Tsai–Hill, Tsai–Wu, Hashin and Puck–Schürmann were discussed more deeply, as they are the most commonly used.

In the ensuing section, the types of elements and theories that govern these were discussed, as were their differences and applications in the simulation of composites. Among all types of available elements, the elements of plate, shell and cohesive were highlighted, since these are the most commonly used.

The last section presents reports from companies and industrial sectors, such as aeronautics, aerospace, automotive, naval, energy, civil, sports, manufacturing and even electronics, which use finite element simulations with composite materials in very different cases. These cases start from simple mechanical test simulations, and go to more complex studies, such as topology optimization; impact; tribology; vibrations; fluid flow; environmental conditioning; effects of natural events (earthquake, tornado and drilling); prediction of damages resulting from the machining; and even electrical properties, such as electrical conduction and capacitance. That came together to demonstrate the great importance that simulations with finite elements of composite materials has presented in recent years.

## Figures and Tables

**Table 1:** Most common failure criteria for composite materials.

Failure Criteria	Formula	References
Maximum Stress	$-X_{c}<\sigma_{1}<X_{t}$; $-Y_{c}<\sigma_{2}<Y_{t}$; $\left|\tau_{12}\right|<S$	[32,65,72,83,89,90,91,92,93,94,95,96,97,98,99,100,101,102,103,104,105,106,107,108,109]
Tsai–Hill	$\frac{\sigma_{1}^{2}}{X^{2}}+\frac{\sigma_{2}^{2}}{Y^{2}}-\frac{\sigma_{1}\;\sigma_{2}}{X^{2}}+\frac{\tau_{12}^{2}}{S^{2}}\leq 1$	[26,28,82,85,87,105,106,110,111,112,113,114,115,116,117,118,119,120,121,122,123,124]
Tsai–Wu	$F_{1}\sigma_{1}+F_{2}\sigma_{2}+F_{6}\tau_{12}+F_{11}\sigma_{1}^{2}+F_{22}\sigma_{2}^{2}+F_{66}\tau_{12}^{2}+2F_{12}\sigma_{1}\sigma_{2}+F_{16}\sigma_{1}\tau_{12}+F_{26}\sigma_{2}\tau_{12}\leq 1$	[82,83,85,87,106,111,122,125,126,127,128,129,130,131,132,133,134,135,136,137,138,139,140,141,142,143,144,145,146,147]
Hashin	${\left(\frac{\sigma_{1}}{X_{T}}\right)}^{2}+{\left(\frac{\tau_{12}}{S_{12}}\right)}^{2}=1$;$\;{\left(\frac{\sigma_{2}}{Y_{T}}\right)}^{2}+{\left(\frac{\tau_{12}}{S_{12}}\right)}^{2}=1$; $\sigma_{1}=-X_{c}$; ${\left(\frac{\sigma_{2}}{2S_{23}}\right)}^{2}+\left[{\left(\frac{Y_{C}}{2S_{23}}\right)}^{2}-1\right]\frac{\sigma_{2}}{Y_{C}}+{\left(\frac{\tau_{12}}{S_{12}}\right)}^{2}=1$	[24,83,85,87,93,104,105,107,122,127,148,149,150,151,152,153,154,155,156,157,158,159,160,161,162,163]
Puck–Schürmann	$\emptyset_{MC}={\left(\frac{t_{L}}{S_{L}-\eta_{L}t_{N}}\right)}^{2}+{\left(\frac{t_{T}}{S_{T}-\eta_{T}t_{N}}\right)}^{2}$	[84,106,155,164,165,166,167,168,169,170,171,172,173,174,175,176,177,178]

## References

[1] Rezende M.C., Costa M.L., Botelho E.C. (2011). Compósitos Estruturais: Tecnologia e Prática.

[2] Callister Junior W.D., Rethwisch D.G. (2013). Materials Science and Engineering: An. Introduction.

[3] Kaw A.K. (2006). Mechanics of Composite Materials.

[4] Durand L.P. (2008). Composite Materials Research Progress.

[5] Chung D.D.L. (2010). Composite Materials. Engineering Materials and Processes.

[6] Levy Neto F., Pardini L.C. (2016). Compósitos Estruturais: Ciência e Tecnologia.

[7] Berthelot J.-M. (1999). Composite Materials: Mechanical Behavior and Structural Analysis.

[8] Gay D. (2014). Composite Materials: Design and Applications.

[9] Teti R. (2002). Machining of Composite Materials. CIRP Ann..

[10] Gay D., Hoa S.V., Tsai S.W. (2003). Composite Materials: Design and Applications.

[11] Li Y., Li W., Lin X., Yang M., Zhao Z., Zhang X., Dong P., Xu N., Sun Q., Dai Y. (2019). Theoretical modeling of the temperature dependent tensile strength for particulate-polymer composites. Compos. Sci. Technol..

[12] Liu D., Tang Y., Cong W.L. (2012). A review of mechanical drilling for composite laminates. Compos. Struct..

[13] Xu J., Mkaddem A., El Mansori M. (2016). Recent advances in drilling hybrid FRP/Ti composite: A state-of-the-art review. Compos. Struct..

[14] Katsoulis C., Kandola B.K., Myler P., Kandare E. (2012). Post-fire flexural performance of epoxy-nanocomposite matrix glass fibre composites containing conventional flame retardants. Compos. Part A Appl. Sci. Manuf..

[15] Matadi Boumbimba R., Froustey C., Viot P., Olive J.M., Léonardi F., Gerard P., Inoubli R. (2014). Preparation and mechanical characterisation of laminate composites made of glass fibre/epoxy resin filled with tri bloc copolymers. Compos. Struct..

[16] Xing J., Du C., He X., Zhao Z., Zhang C., Li Y. (2019). Finite Element Study on the Impact Resistance of Laminated and Textile Composites. Polymers.

[17] Jia Z., Bai Y., Wang F., Ma J., Cheng D., Zhang Z. (2019). Effect of drill flute direction on delamination at the exit in drilling Carbon Fiber Reinforced Plastic. Polym. Compos..

[18] Moura M.F.S.F., Morais A.B., Magalhães A.G. (2005). Materiais Compósitos: Materiais, Fabrico e Comportamento Mecânico.

[19] Ghafoori E., Prinz G., Mayor E., Nussbaumer A., Motavalli M., Herwig A., Fontana M. (2014). Finite Element Analysis for Fatigue Damage Reduction in Metallic Riveted Bridges Using Pre-Stressed CFRP Plates. Polymers.

[20] Martinelli E., Napoli A., Nunziata B., Realfonzo R. (2014). RC Beams Strengthened with Mechanically Fastened Composites: Experimental Results and Numerical Modeling. Polymers.

[21] Cook R.D. (1995). Finite Element Modeling for Stress Analysis.

[22] Tenek L.T., Argyris J., Gladwell G.M. (1998). Finite Element Analysis for Composite Structures. Solid Mechanics and Its Applications.

[23] Dandekar C.R., Shin Y.C. (2012). Modeling of machining of composite materials: A review. Int. J. Mach. Tools Manuf..

[24] Lasri L., Nouari M., El Mansori M. (2009). Modelling of chip separation in machining unidirectional FRP composites by stiffness degradation concept. Compos. Sci. Technol..

[25] Zhao L.G., Warrior N.A., Long A.C. (2006). Finite element modelling of damage progression in non-crimp fabric reinforced composites. Compos. Sci. Technol..

[26] Arola D., Ramulu M. (1997). Orthogonal cutting of fiber-reinforced composites: A finite element analysis. Int. J. Mech. Sci..

[27] Barbero E.J. (2014). Finite Element Analysis of Composite Materials Using Ansys.

[28] Mkaddem A., Demirci I., Mansori M. (2008). El A micro–macro combined approach using FEM for modelling of machining of FRP composites: Cutting forces analysis. Compos. Sci. Technol..

[29] Yang L., Yan Y., Liu Y., Ran Z. (2012). Microscopic failure mechanisms of fiber-reinforced polymer composites under transverse tension and compression. Compos. Sci. Technol..

[30] Venu Gopala Rao G., Mahajan P., Bhatnagar N. (2007). Machining of UD-GFRP composites chip formation mechanism. Compos. Sci. Technol..

[31] Jones R.M. (1998). Mechanics of Composite Materials.

[32] Hinton M.J., Kaddour A.S., Soden P.D. (2004). Failure Criteria in Fibre-Reinforced-Polymer Composites.

[33] Tan W., Naya F., Yang L., Chang T., Falzon B.G., Zhan L., Molina-Aldareguía J.M., González C., Llorca J. (2018). The role of interfacial properties on the intralaminar and interlaminar damage behaviour of unidirectional composite laminates: Experimental characterization and multiscale modelling. Compos. Part B Eng..

[34] Aboudi J., Arnold S.M., Bednarcyk B.A. (2013). Micromechanics of Composite Materials.

[35] Ma J., Du W., Gao W., Wriggers P., Xue X. (2018). Multiscale finite element analysis of uncertain-but-bounded heterogeneous materials at finite deformation. Finite Elem. Anal. Des..

[36] Li D., Wang Z., Zhang C. (2019). A multi-level and multi-site mesh refinement method for the 2D problems with microstructures. Mech. Adv. Mater. Struct..

[37] Elliott J.A. (2011). Novel approaches to multiscale modelling in materials science. Int. Mater. Rev..

[38] Koloor S.S.R., Rahimian-Koloor S.M., Karimzadeh A., Hamdi M., Petrů M., Tamin M.N. (2019). Nano-Level Damage Characterization of Graphene/Polymer Cohesive Interface under Tensile Separation. Polymers.

[39] Wang Z.-X., Shen H.-S. (2012). Nonlinear vibration and bending of sandwich plates with nanotube-reinforced composite face sheets. Compos. Part B Eng..

[40] Wang Z.-X., Shen H.-S. (2011). Nonlinear vibration of nanotube-reinforced composite plates in thermal environments. Comput. Mater. Sci..

[41] Bostanabad R., Liang B., Gao J., Liu W.K., Cao J., Zeng D., Su X., Xu H., Li Y., Chen W. (2018). Uncertainty quantification in multiscale simulation of woven fiber composites. Comput. Methods Appl. Mech. Eng..

[42] Stier B., Simon J.-W., Reese S. (2015). Numerical and experimental investigation of the structural behavior of a carbon fiber reinforced ankle-foot orthosis. Med. Eng. Phys..

[43] Mao J.Z., Sun X.S., Ridha M., Tan V.B.C., Tay T.E. (2013). A Modeling Approach Across Length Scales for Progressive Failure Analysis of Woven Composites. Appl. Compos. Mater..

[44] Yin H., Zhao Y. (2016). Introduction to the Micromechanics of Composite Materials.

[45] Shaik A., Salvi A. (2017). A Multi Scale Approach for Analysis of Fiber Reinforced Composites. Mater. Today Proc..

[46] Mustafa G., Suleman A., Crawford C. (2015). Probabilistic micromechanical analysis of composite material stiffness properties for a wind turbine blade. Compos. Struct..

[47] Kwon Y.W., Allen D.H., Talreja R. (2008). Multiscale Modeling and Simulation of Composite Materials and Structures.

[48] Linde P., Pleitner J., De Boer H., Carmone C. Modelling and Simulation of Fibre Metal Laminates. Proceedings of the ABAQUS Users’ Conference.

[49] Tian W., Qi L., Zhou J., Liang J., Ma Y. (2015). Representative volume element for composites reinforced by spatially randomly distributed discontinuous fibers and its applications. Compos. Struct..

[50] Lu Z., Yuan Z., Liu Q. (2014). 3D numerical simulation for the elastic properties of random fiber composites with a wide range of fiber aspect ratios. Comput. Mater. Sci..

[51] Kanit T., Forest S., Galliet I., Mounoury V., Jeulin D. (2003). Determination of the size of the representative volume element for random composites: Statistical and numerical approach. Int. J. Solids Struct..

[52] Li S., Sitnikova E. (2020). Representative Volume Elements and Unit Cells.

[53] Feito N., Díaz-Álvarez J., López-Puente J., Miguelez M.H. (2018). Experimental and numerical analysis of step drill bit performance when drilling woven CFRPs. Compos. Struct..

[54] Ferreira A.J.M., Viola E., Tornabene F., Fantuzzi N. (2017). Mechcomp3 3rd International Conference of Mechanics of Composite.

[55] Chandra N., Li H., Shet C., Ghonem H. (2002). Some issues in the application of cohesive zone models for metal–ceramic interfaces. Int. J. Solids Struct..

[56] Shojaei A.K., Shao J. (2017). Porous Rock Fracture Mechanics: With Application to Hydraulic Fracturing, Drilling and Structural Engineering.

[57] Milne I., Ritchie R.O., Karihaloo B. (2003). Comprehensive Satructural Integrety.

[58] Beaumont P.W.R., Soutis C., Hodzic A. (2015). Structural Integrity and Durability of Advanced Composites: Innovative Modelling Methods and Intelligent Design.

[59] De Morais A.B. (2013). Mode I cohesive zone model for delamination in composite beams. Eng. Fract. Mech..

[60] Abena A., Soo S.L., Essa K. (2017). Modelling the orthogonal cutting of UD-CFRP composites: Development of a novel cohesive zone model. Compos. Struct..

[61] Yang Q., Cox B. (2005). Cohesive models for damage evolution in laminated composites. Int. J. Fract..

[62] Xie J., Waas A.M., Rassaian M. (2016). Closed-form solutions for cohesive zone modeling of delamination toughness tests. Int. J. Solids Struct..

[63] Dávila C.G., Camanho P.P., Turon A. (2007). Cohesive Elements for Shells.

[64] Harper P.W., Hallett S.R. (2008). Cohesive zone length in numerical simulations of composite delamination. Eng. Fract. Mech..

[65] Vassilopoulos A.P. (2015). Fatigue and Fracture of Adhesively-Bonded Composite Joints.

[66] Katnam K.B., Sargent J.P., Crocombe A.D., Khoramishad H., Ashcroft I.A. (2010). Characterisation of moisture-dependent cohesive zone properties for adhesively bonded joints. Eng. Fract. Mech..

[67] Khoramishad H., Crocombe A.D., Katnam K.B., Ashcroft I.A. (2010). Predicting fatigue damage in adhesively bonded joints using a cohesive zone model. Int. J. Fatigue.

[68] Liu P.F., Zheng J.Y. (2010). Recent developments on damage modeling and finite element analysis for composite laminates: A review. Mater. Des..

[69] Graham-Jones J., Summerscales J. (2016). Marine Applications of Advanced Fibre-Reinforced Composites.

[70] Tillmann V.B. (2015). Análise Estrutural de Elementos Compósitos Com a Utilização do Método de Elementos Finitos.

[71] Azevedo C.A.C. (2007). de Formulação Alternativa para Análise de Domínios Não-Homogêneos e Inclusões Anisotrópicas via Mec.

[72] Daniel I. (2006). Engineering Mechanics of Composites Material.

[73] Bednarcyk B.A., Aboudi J., Arnold S.M. (2017). Micromechanics of composite materials governed by vector constitutive laws. Int. J. Solids Struct..

[74] Bauchau O.A., Craig J.I. (2009). Structural Analysis. Solid Mechanics and Its Applications.

[75] Soriano H.L. (2003). Método de Elementos Finitos em Análise de Estruturas.

[76] Vanalli L. (2004). O MEC e o MEF Aplicados a Análise de Problemas Viscoplasticos em Meios Anisotrópicos e Compostos.

[77] Barbero E.J. (2008). Finite Element Analysis of Composite Materials.

[78] Ochoa O.O., Reddy J.N., Gladwell G.M.L. (1992). Finite Element Analysis of Composite Laminates. Solid Mechanics and Its Applications.

[79] Ojo S.O., Ismail S.O., Paggi M., Dhakal H.N. (2017). A new analytical critical thrust force model for delamination analysis of laminated composites during drilling operation. Compos. Part. B Eng..

[80] Feng W.W. (1991). A Failure Criterion for Composite Materials. J. Compos. Mater..

[81] Mendonça P.D.T.R. (2005). Materiais Compostos & Estruturas-Sanduíche: Projetos e Análises.

[82] Zhao S.Y., Xue P. (2014). New Two-Dimensional Polynomial Failure Criteria for Composite Materials. Adv. Mater. Sci. Eng..

[83] Daniel I.M., Werner B.T., Fenner J.S. (2011). Strain-rate-dependent failure criteria for composites. Compos. Sci. Technol..

[84] Ashouri Vajari D. (2015). A micromechanical study of porous composites under longitudinal shear and transverse normal loading. Compos. Struct..

[85] Paris F. (2001). A Study of Failure Criteria of Fibrous Composite Materials.

[86] Qing H., Mishnaevsky L. (2010). 3D constitutive model of anisotropic damage for unidirectional ply based on physical failure mechanisms. Comput. Mater. Sci..

[87] Yeh H.-Y., Murphy H.C., Yeh H.-L. (2009). An Investigation of Failure Criterion for New Orthotropic Ceramic Matrix Composite Materials. J. Reinf. Plast. Compos..

[88] Camanho P.P., Dávila C.G. (2002). Mixed-Mode Decohesion Finite Elements for the Simulation of Delamination in Composite Materials.

[89] Shokrieh M.M. (2014). Residual Stresses in Composite Materials.

[90] Vasiliev V.V., Morozov E.V. (2013). Advanced Mechanics of Composite Materials.

[91] Martiny P., Lani F., Kinloch A.J., Pardoen T. (2013). A maximum stress at a distance criterion for the prediction of crack propagation in adhesively-bonded joints. Eng. Fract. Mech..

[92] Bobrov A.V., Sarbaev B.S., Shirshov Y.Y. (2016). Deformation and strength properties of a carbon–carbide composite with 2D reinforcement under plane stress state. J. Mach. Manuf. Reliab..

[93] Jiang H., Ren Y., Liu Z., Zhang S., Wang X. (2018). Evaluations of failure initiation criteria for predicting damages of composite structures under crushing loading. J. Reinf. Plast. Compos..

[94] Promis G., Bach T.Q., Gabor A., Hamelin P. (2014). Failure behavior of E-glass fiber- and fabric-reinforced IPC composites under tension and compression loading. Mater. Struct..

[95] Pyttel T., Liebertz H., Cai J. (2011). Failure criterion for laminated glass under impact loading and its application in finite element simulation. Int. J. Impact Eng..

[96] Chang K.J. (1981). Further studies of the maximum stress criterion on the angled crack problem. Eng. Fract. Mech..

[97] Luo C., Xiong J. (2012). Static Pull and Push Bending Properties of RTM-made TWF Composite Tee-joints. Chin. J. Aeronaut..

[98] Hart-Smith L. (1998). Predictions of a generalized maximum-shear-stress failure criterion for certain fibrous composite laminates. Compos. Sci. Technol..

[99] Hiroshima N., Hatta H., Koyama M., Goto K., Kogo Y. (2015). Optimization of flywheel rotor made of three-dimensional composites. Compos. Struct..

[100] Raftery G.M., Harte A.M. (2013). Nonlinear numerical modelling of FRP reinforced glued laminated timber. Compos. Part B Eng..

[101] Zhao L., Qin T., Zhang J., Shenoi R.A. (2013). Modified maximum stress failure criterion for composite π joints. J. Compos. Mater..

[102] Feng P., Meng X., Chen J.-F., Ye L. (2015). Mechanical properties of structures 3D printed with cementitious powders. Constr. Build. Mater..

[103] Koerber H., Xavier J., Camanho P.P., Essa Y.E., Martín de la Escalera F. (2015). High strain rate behaviour of 5-harness-satin weave fabric carbon–epoxy composite under compression and combined compression–shear loading. Int. J. Solids Struct..

[104] Echaabi J., Trochu F., Gauvin R. (1996). Review of Failure Criteria of Fibrous Composite Materials. Polym. Compos..

[105] Ferreira L.B.M., Júniora R.C.S.F., Forda E.T.L.C. (2019). Analysis of failure criteria in laminas reinforced with unidirectional Curaua fiber fabric. Mater. Res..

[106] De Luca A., Caputo F. (2017). A review on analytical failure criteria for composite materials. AIMS Mater. Sci..

[107] Ataş A. (2015). Open hole compressive strength and damage mechanisms: Maximum stress versus Hashin criteria. Plast. Rubber Compos..

[108] Vasiliev V.V., Morozov E.V. (2018). Advanced Mechanics of Composite Materials and Structures.

[109] Barbero E.J. (2018). Introduction to Composite Materials Design.

[110] Mkaddem A., El Mansori M. (2009). Finite element analysis when machining UGF-reinforced PMCs plates: Chip formation, crack propagation and induced-damage. Mater. Des..

[111] Yang C., Jiao G., Guo H. (2014). Failure criteria for C/SiC composites under plane stress state. Theor. Appl. Mech. Lett..

[112] Mohamadi M., Heshmati M. (2019). Failure analysis of glass-reinforced polyester mortar pipes with different cores subjected to combined loading. J. Sandw. Struct. Mater..

[113] Fragoudakis R. (2019). A numerical approach to determine fiber orientations around geometric discontinuities in designing against failure of GFRP laminates. Int. J. Struct. Integr..

[114] Rao P.M., Walter T.R., Sankar B., Subhash G., Yen C.F. (2014). Analysis of failure modes in three-dimensional woven composites subjected to quasi-static indentation. J. Compos. Mater..

[115] Heidari-Rarani M., Khalkhali-Sharifi S.S., Shokrieh M.M. (2014). Effect of ply stacking sequence on buckling behavior of E-glass/epoxy laminated composites. Comput. Mater. Sci..

[116] Garmsiri K., Jalal M. (2014). Multiobjective optimization of composite cylindrical shells for strength and frequency using genetic algorithm and neural networks. Sci. Eng. Compos. Mater..

[117] Liu C. (2019). Reliability Analysis of Composite Laminates Based on Generating Function Approach. J. Mech. Eng..

[118] Morgado T., Silvestre N., Correia J.R. (2019). Simulation of fire resistance behaviour of pultruded GFRP columns. Thin Walled Struct..

[119] Naik N.K., Sekher Y.C. (1998). Damage in Laminated Composites Due to Low Velocity Impact. J. Reinf. Plast. Compos..

[120] Singh S., Kumar A. (1998). Postbuckling response and failure of symmetric laminates under in-plane shear. Compos. Sci. Technol..

[121] Jen M. (1998). Strength and life in thermoplastic composite laminates under static and fatigue loads. Part I: Experimental. Int. J. Fatigue.

[122] Daniel I.M. (2016). Yield and failure criteria for composite materials under static and dynamic loading. Prog. Aerosp. Sci..

[123] Rohwer K. (2015). Predicting fiber composite damage and failure. J. Compos. Mater..

[124] Rao G.V.G., Mahajan P., Bhatnagar N. (2008). Three-dimensional macro-mechanical finite element model for machining of unidirectional-fiber reinforced polymer composites. Mater. Sci. Eng. A.

[125] Tsai S.W., Hahn H.T. (1980). Introduction to Composite Materials.

[126] Guidi E.S., de Azevedo Silva F. Study of Stress Concentration in Polymeric Composites Using Finite Element Method. Proceedings of the SAE Technical Papers.

[127] Guidi E.S., de Azevedo Silva F. Analysis of mechanical behavior in bending of polymer composites using the finite elements method. Proceedings of the SAE Technical Papers.

[128] Takkar S., Gupta K., Tiwari V., Singh S.P. (2019). Dynamics of Rotating Composite Disc. J. Vib. Eng. Technol..

[129] Al-Saadi A., Aravinthan T., Lokuge W., Wang C., Ho J., Kitipornchai S. (2020). Numerical Investigation on Hollow Pultruded Fibre Reinforced Polymer Tube Columns. ACMSM25: Lecture Notes in Civil Engineering.

[130] Turan K. (2013). Joint angle effect on the failure behavior of pinned joint composite plates. J. Compos. Mater..

[131] Muthusamy P., Sivakumar S.M. (2014). A constituent-behavior-motivated model for damage in fiber reinforced composites. Comput. Mater. Sci..

[132] Rajpal D., Kassapoglou C., De Breuker R. (2019). Aeroelastic optimization of composite wings including fatigue loading requirements. Compos. Struct..

[133] Imran M., Shi D., Tong L., Waqas H.M. (2019). Design optimization of composite submerged cylindrical pressure hull using genetic algorithm and finite element analysis. Ocean Eng..

[134] Fathallah E., Qi H., Tong L., Helal M. (2015). Design optimization of lay-up and composite material system to achieve minimum buoyancy factor for composite elliptical submersible pressure hull. Compos. Struct..

[135] Cousigné O., Moncayo D., Coutellier D., Camanho P., Naceur H., Hampel S. (2013). Development of a new nonlinear numerical material model for woven composite materials accounting for permanent deformation and damage. Compos. Struct..

[136] Hu H.-T., Lin W.-P., Tu F.-T. (2015). Failure analysis of fiber-reinforced composite laminates subjected to biaxial loads. Compos. Part B Eng..

[137] Sulaiman S., Borazjani S., Tang S.H. (2013). Finite element analysis of filament-wound composite pressure vessel under internal pressure. IOP Conf. Ser. Mater. Sci. Eng..

[138] Osei-Antwi M., de Castro J., Vassilopoulos A.P., Keller T. (2014). Fracture in complex balsa cores of fiber-reinforced polymer sandwich structures. Constr. Build. Mater..

[139] Liu X., Gasco F., Goodsell J., Yu W. (2019). Initial failure strength prediction of woven composites using a new yarn failure criterion constructed by deep learning. Compos. Struct..

[140] Li W., Cai H., Li C., Wang K., Fang L. (2015). Micro-mechanics of failure for fatigue strength prediction of bolted joint structures of carbon fiber reinforced polymer composite. Compos. Struct..

[141] Helal M., Huang H., Wang D., Fathallah E. (2019). Numerical Analysis of Sandwich Composite Deep Submarine Pressure Hull Considering Failure Criteria. J. Mar. Sci. Eng..

[142] Debski H., Teter A. (2015). Numerical and experimental studies on the limit state of fibre-reinforced composite columns with a lipped channel section under quasi-static compression. Compos. Struct..

[143] Sun E., Ghoshal P.K., Fair R.J., Lassiter S.R., Brindza P. (2020). Quench-Back Management for Fast Decaying Currents in SHMS Superconducting Magnets at Jefferson Lab. IEEE Trans. Appl. Supercond..

[144] Vignoli L.L., de Castro J.T.P., Meggiolaro M.A. (2019). Stress concentration issues in unidirectional laminates. J. Brazilian Soc. Mech. Sci. Eng..

[145] Tsai S.W., Wu E.M. (1971). A General Theory of Strength for Anisotropic Materials. J. Compos. Mater..

[146] Siqueira E.J., Silva F.A., Botelho E.C. (2017). Estudo dos Critérios de Falhas em Compósitos PPS/Fibras de Carbono Utilizando Elementos Finitos. Epistem. Transversalis.

[147] Kawai M., Morishita M., Tomura S., Takumida K. (1998). Inelastic behavior and strength of fiber-metal hybrid composite: Glare. Int. J. Mech. Sci..

[148] Hashin Z., Herakovich C.T. (1983). Mechanics of Composite Materials.

[149] Hashin Z. (1981). Fatigue Failure Criteria for Unidirectional Fiber Composites. J. Appl. Mech..

[150] Hashin Z. (1980). Failure Criteria for Unidirectional Fiber Compsites. J. Appl. Mech..

[151] Isbilir O., Ghassemieh E. (2014). Three-dimensional numerical modelling of drilling of carbon fiber-reinforced plastic composites. J. Compos. Mater..

[152] Isbilir O., Ghassemieh E. (2013). Numerical investigation of the effects of drill geometry on drilling induced delamination of carbon fiber reinforced composites. Compos. Struct..

[153] Fan X.L., Wang T.J., Sun Q. (2011). Damage evolution of sandwich composite structure using a progressive failure analysis methodology. Procedia Eng..

[154] Guillaumat L., Hamdoun Z. (2006). Reliability model of drilled composite materials. Compos. Struct..

[155] Cepero-Mejias F., Phadnis V.A., Curiel-Sosa J.L. (2019). Machining induced damage in orthogonal cutting of UD composites: FEA based assessment of Hashin and Puck criteria. Procedia CIRP.

[156] Larbi Chaht F., Mokhtari M., Benzaama H. (2019). Using a Hashin Criteria to predict the Damage of composite notched plate under traction and torsion behavior. Frattura Integrità Strutt..

[157] Santiuste C., Sánchez-Sáez S., Barbero E. (2010). A comparison of progressive-failure criteria in the prediction of the dynamic bending failure of composite laminated beams. Compos. Struct..

[158] Ghannadpour S.A.M., Kurkaani Barvaj A., Tornabene F. (2018). A semi-analytical investigation on geometric nonlinear and progressive damage behavior of relatively thick laminated plates under lateral pressure and end-shortening. Compos. Struct..

[159] Hamza K., Shash A.Y., Abdrabou M. (2018). Design criteria of multilayer fibers reinforced composite in bulky 3D loaded applications. Compos. Part B Eng..

[160] Kim D.-H., Kim S.-W. (2019). Evaluation of bird strike-induced damages of helicopter composite fuel tank assembly based on fluid-structure interaction analysis. Compos. Struct..

[161] Kress G. (2013). Examination of Hashin’s failure criteria for Part B of the second world-wide failure exercise: Comparison with test data. J. Compos. Mater..

[162] Kress G. (2012). Examination of Hashin’s failure criteria for the second world-wide failure exercise. J. Compos. Mater..

[163] Gu J., Chen P. (2017). Some modifications of Hashin’s failure criteria for unidirectional composite materials. Compos. Struct..

[164] Thomson D.M., Cui H., Erice B., Hoffmann J., Wiegand J., Petrinic N. (2017). Experimental and numerical study of strain-rate effects on the IFF fracture angle using a new efficient implementation of Puck’s criterion. Compos. Struct..

[165] Wang Z., Zhao J. (2019). Low velocity impact response of GLARE laminates based on a new efficient implementation of Puck’s criterion. Thin Walled Struct..

[166] Talreja R., Varna J. (2016). Modeling Damage, Fatigue and Failure of Composite Materials.

[167] Puck A., Schürmann H. (2002). Failure analysis of FRP laminates by means of physically based phenomenological models. Compos. Sci. Technol..

[168] De Macedo R.Q., Ferreira R.T.L., Guedes J.M., Donadon M.V. (2017). Intraply failure criterion for unidirectional fiber reinforced composites by means of asymptotic homogenization. Compos. Struct..

[169] De Macedo R.Q., Guedes J.M., Luiz Ferreira R.T., Donadon M.V. A failure criteria for unidirectional fiber reinforced composites based on micromechanics by asymptotic homogenization. Proceedings of the VII European Congress on Computational Methods in Applied Sciences and Engineering.

[170] Deuschle H.M., Puck A. (2013). Application of the Puck failure theory for fibre-reinforced composites under three-dimensional stress: Comparison with experimental results. J. Compos. Mater..

[171] Puck A., Deuschle H.M. (2002). Progress in the puck failure theory for fibre reinforced composites: Analytical solutions for 3D-stress. Compos. Sci. Technol..

[172] Kim S.-Y., Shim C.S., Sturtevant C., Kim D., Song H.C. (2014). Mechanical properties and production quality of hand-layup and vacuum infusion processed hybrid composite materials for GFRP marine structures. Int. J. Nav. Archit. Ocean Eng..

[173] Soden P. (1998). A comparison of the predictive capabilities of current failure theories for composite laminates. Compos. Sci. Technol..

[174] Schirmaier F.J., Weiland J., Kärger L., Henning F. (2014). A new efficient and reliable algorithm to determine the fracture angle for Puck’s 3D matrix failure criterion for UD composites. Compos. Sci. Technol..

[175] Gadade A.M., Lal A., Singh B.N. (2016). Accurate stochastic initial and final failure of laminated plates subjected to hygro-thermo-mechanical loadings using Puck’s failure criteria. Int. J. Mech. Sci..

[176] Puck A., Schürmann H., Hinton M.J., Kaddour A.S., Soden P.D. (2004). Failure analysis of FRP laminates by means of physically based phenomenological models. Failure Criteria in Fibre-Reinforced-Polymer Composites.

[177] Puck A., Schürmann H. (1998). Failure analysis of FRP laminates by means of physically based phenomenological models. Compos. Sci. Technol..

[178] Cepero-Mejías F., Curiel-Sosa J.L., Zhang C., Phadnis V.A. (2019). Effect of cutter geometry on machining induced damage in orthogonal cutting of UD polymer composites: FE study. Compos. Struct..

[179] Arouche M.M. (2015). Estudo Numérico-Experimental de Materiais Compósitos Utilizados no Reparo de Estruturas Navais.

[180] Daniel I.M., Ishai O. (2005). Engineering mechanics of composite materials.

[181] Tita V., Carvalho J., Santos N.C. Estudo do comportamento mecânico de materiais compósitos utilizando o método dos elementos finitos. Proceedings of the II Congresso Nacional de Engenharia Mecânica–Conem.

[182] Diniz C.A. (2017). Otimização Estrutural de Elementos em Compósitos Usando Redes Neurais Artificiais.

[183] Carvalho G.P. (2003). de Avaliação de Critérios de Falhas de Compósitos Poliméricos Reforçados Aplicados a Vigas sob Carregamento de Flexão.

[184] Gagliardo D.P. (2008). Análise de Estruturas Sanduíche: Parâmetros de Projeto.

[185] Campos V.B.O. (2017). Análise de Critérios de Falha em Lâmina Reforçada com Tecido Híbrido de Fibra de Vidro e Carbono.

[186] Laurin F., Carrere N., Maire J.-F., Camanho P.P., Hallett S.R. (2015). Strength prediction methods for composite structures. Numerical Modelling of Failure in Advanced Composite Materials.

[187] Altenbach H., Sadowski T. (2015). Failure and Damage Analysis of Advanced Materials.

[188] Khan A., Johnson E., Kapania R., Batra R., Guimard J.-M. Progressive Failure Analysis of Laminated Composite Structures Based on Puck’s Failure Criteria. Proceedings of the 53rd AIAA/ASME/ASCE/AHS/ASC Structures, Structural Dynamics and Materials Conference.

[189] Dávila C.G., Camanho P.P., Rose C.A. (2005). Failure criteria for FRP laminates. J. Compos. Mater..

[190] Deuschle H.M. (2010). 3D Failure Analysis of UD Fibre Reinforced Composites: Puck’s Theory within FEA.

[191] Daniel I.M., Luo J.J., Schubel P.M., Werner B.T. (2009). Interfiber/interlaminar failure of composites under multi-axial states of stress. Compos. Sci. Technol..

[192] Zienkiewicz O.C., De J.P., Kelly D.W. (1983). The hierarchical concept in finite element analysis. Comput. Struct..

[193] Zienkiewicz O.C., Emeritus F. (2000). The Finite Element Method: The Basis.

[194] Cheng Z.-Q., Batra R.C. (2000). Deflection relationships between the homogeneous Kirchhoff plate theory and different functionally graded plate theories. Arch. Mech..

[195] Azevedo F.M. (2003). Método dos Elementos Finitos.

[196] Matthews F.L., Davies G.A.O., Hitchings D., Soutis C. (2003). Finite Element Modelling of Composite Materials and Structures.

[197] Zienkiewicz O.C., Emeritus F. (2000). The Finite Element Method: Solid Mechanics.

[198] Adams V., Askenazi A. (1999). Building Better Products with Finit Elements Analysis.

[199] Ugural A.C. (1981). Stresses in Plates and Shells.

[200] Timoshenko S., Woinowsky-Krieger S. (1959). Thoery of Plates and Shells.

[201] Vaz L.E. (2011). Método dos Elementos Finitos em Análise de Estruturas.

[202] Valsiliev V.V., Morozov E.V. (2001). Mechanics and Analysis of Composite Materials.

[203] Altenbach H., Eremeyev V. (2017). Shell-Like Structures.

[204] Tsiatas G.C. (2009). A new Kirchhoff plate model based on a modified couple stress theory. Int. J. Solids Struct..

[205] Phadnis V.A., Roy A., Silberschmidt V.V. (2012). Finite element analysis of drilling in carbon fiber reinforced polymer composites. J. Phys. Conf. Ser..

[206] Nguyen-Xuan H., Rabczuk T., Bordas S., Debongnie J.F. (2008). A smoothed finite element method for plate analysis. Comput. Methods Appl. Mech. Eng..

[207] Chai Y.B., Li W., Gong Z.X. An edge-based smoothed three-node Mindlin plate element (ES-MIN3) for static. Proceedings of the 5th International Conference on Computational Methods.

[208] Zienkiewicz O.C., Taylor R.L., Too J.M. (1971). Reduced integration technique in general analysis of plates and shells. Int. J. Numer. Methods Eng..

[209] Altenbach J., Altenbach H., Eremeyev V.A. (2010). On generalized Cosserat-type theories of plates and shells: A short review and bibliography. Arch. Appl. Mech..

[210] Lu P., He L.H., Lee H.P., Lu C. (2006). Thin plate theory including surface effects. Int. J. Solids Struct..

[211] Norrie D.H., Vries G. (1973). de The Finite Element Method: Fundamentals and Applications.

[212] Saliba S.S., Penna S.S., Pitangueira R.L. (2011). Abordagem Orientada a Objetos Para Implementação Computacional de Elementos Finitos de PLacas. Cad. Eng. Estrut..

[213] Schneider P., Kienzler R., Böhm M. (2014). Modeling of consistent second-order plate theories for anisotropic materials. ZAMM Z. Angew. Math. Mech..

[214] Saliba S.S. (2007). Implementação Computacional e Análise Crítica de Elementos Finitos de Placas.

[215] Ota N.S.N. (2016). O Elemento Finito T6-3I na Análise de Placas e Dinâmica de Cascas.

[216] Chróścielewski J., Gilewski W., Kreja I. Fifty years of Finite Element Analysis pf plate and shells (1960-2010). Proceedings of the 19th International Conference on Computer Methods in Mechanics.

[217] Gileva L., Shaydurov V., Dobronets B. (2013). The Triangular Hermite Finite Element Complementing the Bogner-Fox-Schmit Rectangle. Appl. Math..

[218] Barik M., Mukhopadhyay M. (2002). A new stiffened plate element for the analysis of arbitrary plates. Thin Walled Struct..

[219] Greimann L.F., Lynn P.P. (1970). Finite Element Analysis of plate Pending with Transversal Sahear Deformation. Nucl. Eng. Des..

[220] Ke L.-L., Wang Y.-S., Yang J., Kitipornchai S. (2012). Free vibration of size-dependent Mindlin microplates based on the modified couple stress theory. J. Sound Vib..

[221] Huang H.-C. (1989). Static and Dynamic Analyses of Plates and Shells: Theory, Software and Applications.

[222] Pietraszkiewicz W., Witkowski W. (2017). Shell Structures: Theory and Applications.

[223] Kreja I., Pietraszkiewicz W. (2010). Shell Structures: Theory and Applications.

[224] Alves A.D.R.S., Leggerini M.R.C. (2011). Análise estrutural de cascas via elementos finitos em concreto armado. Rev. Grad..

[225] Jawad M.H. (1994). Various Applications of the Membrane Theory. Theory and Design of Plate and Shell Structures.

[226] Ferreira A.J.M. (1990). Análise por Elementos Finitos de Estruturas Tipo Casca em Materiais Compósitos.

[227] Jullien J.F. (1991). Buckling of Shell Structures, on Land, in the Sea and in the Air.

[228] Monneau R. (2003). Justification of the Nonlinear Kirchhoff-Love Theory of Plates as the Application of a New Singular Inverse Method. Arch. Ration. Mech. Anal..

[229] Ajeje F.H. (2009). Abordagem orientada a objetos para implementação computacional de elementos finitos de cascas planos.

[230] Pietraszkiewicz W., Górki J. (2013). Shell Structures: Theory and Applications.

[231] Rankin C.C., Regelbrugge M., Hurlbut B. Advanced Decohesion Elements for the Simulation of Composite Delamination. Proceedings of the 2010 SIMULIA Customer Conference.

[232] Simulia Abaqus Analysis User’s Guide: Cohesive Elements. https://abaqus-docs.mit.edu/2017/English/SIMACAEELMRefMap/simaelm-c-cohesiveoverview.htm.

[233] Flores-Johnson E.A., Shen L., Guiamatsia I., Nguyen G.D. (2014). Numerical investigation of the impact behaviour of bioinspired nacre-like aluminium composite plates. Compos. Sci. Technol..

[234] Cabello M., Turon A., Zurbitu J., Renart J., Sarrado C., Martínez F. (2017). Progressive failure analysis of DCB bonded joints using a new elastic foundation coupled with a cohesive damage model. Eur. J. Mech. A/Solids.

[235] Wang G.-D., Melly S.K. (2018). Three-dimensional finite element modeling of drilling CFRP composites using Abaqus/CAE: A review. Int. J. Adv. Manuf. Technol..

[236] Xu X.-P., Needleman A. (1994). Numerical simulations of fast crack growth in brittle solids. J. Mech. Phys. Solids.

[237] Dixit U.S., Kant R. (2018). Simulations for Design and Manufacturing.

[238] Espinha R.S.L. (2011). Suporte Topológico em Paralelo para Malhas de Elementos Finitos em Análises Dinâmicas de Fratura e Fragmentação.

[239] Guilpin A., Franciere G., Barton L., Blacklock M., Birkett M. (2019). A Numerical and experimental study of adhesively- bonded polyethylene pipelines. Polymers (Basel).

[240] Xiong X., Xiao Q. (2019). Meso-Scale Simulation of Concrete Based on Fracture and Interaction Behavior. Appl. Sci..

[241] Murali Krishna M., Shunmugam M.S., Siva Prasad N. (2007). A study on the sealing performance of bolted flange joints with gaskets using finite element analysis. Int. J. Press. Vessel. Pip..

[242] Nelson N.R., Prasad N.S. (2016). Sealing behavior of twin gasketed flange joints. Int. J. Press. Vessel. Pip..

[243] Scheider I. (2009). Micromechanical based derivation of traction-separation laws for cohesive model simulations. Procedia Eng..

[244] Park K., Paulino G.H. (2011). Cohesive Zone Models: A Critical Review of Traction-Separation Relationships Across Fracture Surfaces. Appl. Mech. Rev..

[245] Ridha M., Tan V.B.C., Tay T.E. (2011). Traction–separation laws for progressive failure of bonded scarf repair of composite panel. Compos. Struct..

[246] Heidari-Rarani M., Sayedain M. (2019). Finite element modeling strategies for 2D and 3D delamination propagation in composite DCB specimens using VCCT, CZM and XFEM approaches. Theor. Appl. Fract. Mech..

[247] Hu P., Shi Z.W., Wang X.X., Li W.D., Zhou S.G., Han X. (2015). Strength Degradation of Adhesively Bonded Single-Lap Joints in a Cyclic-Temperature Environment Using a Cohesive Zone Model. J. Adhes..

[248] Arafah D.Z.R. (2014). Fracture Assessment of Cracked Components under Biaxial Loading.

[249] Sun M., Chang M., Wang Z., Li H., Sun X. (2018). Experimental and Simulation Study of Low-Velocity Impact on Glass Fiber Composite Laminates with Reinforcing Shape Memory Alloys at Different Layer Positions. Appl. Sci..

[250] Camanho P.P., Davila C.G., de Moura M.F. (2003). Numerical Simulation of Mixed-Mode Progressive Delamination in Composite Materials. J. Compos. Mater..

[251] Schwalbe K.-H., Scheider I., Cornec A. (2013). Guidelines for Applying Cohesive Models to the Damage Behaviour of Engineering Materials and Structures. Springer Briefs in Applied Sciences and Technology.

[252] Budiman B.A., Takahashi K., Inaba K., Kishimoto K. (2016). Evaluation of interfacial strength between fiber and matrix based on cohesive zone modeling. Compos. Part A Appl. Sci. Manuf..

[253] (2016). Best of Automotive. ANSYS Advant. Mag..

[254] Wong K. Many Composite Layers to Address. https://www.digitalengineering247.com/article/many-composite-layers-to-address/ansys-f1-front-wing-failure-plot/.

[255] Anaglyph Laminate Tools. https://www.anaglyph.co.uk/laminate_tools.htm.

[256] Structural Design & Analysis, I. FIBERSIM: Design, Analyze, and Manufacture Composites. https://structures.aero/software/fibersim/.

[257] Siemens Industry Software Inc. The End-to-End Design and Manufacturing Solution for Composite Parts: FIBERSIM. https://www.plm.automation.siemens.com/global/pt/products/nx/fibersim.html.

[258] Altair Engineering Inc. Products. https://altairhyperworks.com/.

[259] Autodesk Inc. Helius Composite: Composite Material Design Simulation Tools. https://www.autodesk.com/products/helius-composite/overview.

[260] Collier Research Corporation Hyper Sizer. https://hypersizer.com/.

[261] NISA NISA—Composite. https://www.nisasoftware.com/composite.

[262] Donaldson B., Sloan J. (2019). Next-Gen Aerospace: Advanced Materials and Processes. GBM Suppl. Next Gen. Aerosp..

[263] TenCate Advanced Composites North America TenCate Composite Materials Replace Titanium for Orion Space Capsule Heatshield and Backshell Structure. https://www.toraytac.com/company/news/2014/12/5/TenCate-composite-materials-replace-titanium-for-Orion-space-capsule-heatshield-and-backshell-structure.

[264] Laflamme Ingénierie Engineering of Advanced Composite Materials. http://www.laflamme-ing.com/en/engineering-services/engineering-of-composite-materials/%0A.

[265] Dlubal Software GmbH Software de Cálculo Estrutural e Dimensionamento: Software para Análise Estrutural e Dimensionamento de Pontes. https://www.dlubal.com/pt/solucoes/areas/pontes.

[266] Abdel-Nasser Y.A. (2013). Frontal crash simulation of vehicles against lighting columns using FEM. Alexandria Eng. J..

[267] Venås J.V., Kvamsdal T. (2020). Isogeometric boundary element method for acoustic scattering by a submarine. Comput. Methods Appl. Mech. Eng..

[268] Lee K., Han S.E., Hong J.W. (2013). Analysis of impact of large commercial aircraft on a prestressed containment building. Nucl. Eng. Des..

[269] Skvortsov Y.V., Glushkov S.V., Chernyakin S.A. (2017). Space Factors Influence on the Size Stability of Small Spacecraft Structure Elements. Procedia Eng..

[270] Gondaliya R., Sypeck D., Zhu F. Improving Damage Tolerance of Composite Sandwich Structure Subjected to Low Velocity Impact Loading: Experimental Analy: UAsis. Proceedings of the 31st American Society for Composites.

[271] Rayavarapu V. (2016). To the Test. ANSYS Advant. Mag..

[272] Zhou Y., Sun Y., Huang T. (2019). SPH-FEM Design of Laminated Plies under Bird-Strike Impact. Aerospace.

[273] Bouvet C., Rivallant S., Barrau J.J. (2012). Low velocity impact modeling in composite laminates capturing permanent indentation. Compos. Sci. Technol..

[274] Abrate S. (1998). Impact on Composite Structures.

[275] Hongkarnjanakul N., Bouvet C., Rivallant S. (2013). Validation of low velocity impact modelling on different stacking sequences of CFRP laminates and influence of fibre failure. Compos. Struct..

[276] (2017). Carbon Freight Ligten Up. ANSYS Advant. Mag..

[277] Huang Y., Yang Z., Liu A., Fu J. (2018). Nonlinear Buckling Analysis of Functionally Graded Graphene Reinforced Composite Shallow Arches with Elastic Rotational Constraints under Uniform Radial Load. Materials.

[278] Yang Z., Huang Y., Liu A., Fu J., Wu D. (2019). Nonlinear in-plane buckling of fixed shallow functionally graded graphene reinforced composite arches subjected to mechanical and thermal loading. Appl. Math. Model..

[279] Díez-Pascual A.M., Naffakh M., Marco C., Ellis G., Gómez-Fatou M.A. (2012). High-performance nanocomposites based on polyetherketones. Prog. Mater. Sci..

[280] Moniruzzaman M., Winey K.I. (2006). Polymer Nanocomposites Containing Carbon Nanotubes. Macromolecules.

[281] Rahman A., Ali I., Al Zahrani S.M., Eleithy R.H. (2011). A review of the applications of nanocarbon polymer composites. Nano.

[282] Sahoo N.G., Rana S., Cho J.W., Li L., Chan S.H. (2010). Polymer nanocomposites based on functionalized carbon nanotubes. Prog. Polym. Sci..

[283] Carballeira P. (2010). Mechanical and Electrical Properties of Carbon Nanofiber–Ceramic Nanoparticle–Polymer Composites.

[284] Scarponi C. (2015). Hemp fiber composites for the design of a Naca cowling for ultra-light aviation. Compos. Part B Eng..

[285] Meng Y., Yan L., Huang W., Zhang T., Du Z. (2019). Structural design and analysis of a composite wing with high aspect ratio. J. Zhejiang Univ. A.

[286] Tam M., Yang Z., Zhao S., Yang J. (2019). Vibration and Buckling Characteristics of Functionally Graded Graphene Nanoplatelets Reinforced Composite Beams with Open Edge Cracks. Materials.

[287] (2019). Safe and Sound with Simulation. ANSYS Advant. Mag..

[288] Yue K., Liu W., Li G., Ji J., Yu D. (2015). Numerical simulation of RCS for carrier electronic warfare airplanes. Chin. J. Aeronaut..

[289] James K.A., Kennedy G.J., Martins J.R.R.A. (2014). Concurrent aerostructural topology optimization of a wing box. Comput. Struct..

[290] Gad-el-Hak I. (2019). Fluid–Structure Interaction for Biomimetic Design of an Innovative Lightweight Turboexpander. Biomimetics.

[291] Space Structures Spacecraft Structures. https://spacestructures.de/references_sc-struct.php.

[292] Vasiliev V.V., Razin A.F. (2006). Anisogrid composite lattice structures for spacecraft and aircraft applications. Compos. Struct..

[293] Pindado S., Roibas-Millan E., Cubas J., Garcia A., Sanz A., Franchini S., Perez-Grande I., Alonso G., Perez-Alvarez J., Sorribes-Palmer F. The UPMSat-2 Satellite: An Academic Project within Aerospace Engineering Education. Proceedings of the 2nd Annual International Conference.

[294] (2016). Engineering the Internet of Things. ANSYS Advant. Mag..

[295] Capiez-Lernout E., Pellissetti M., Pradlwarter H., Schueller G.I., Soize C. (2006). Data and model uncertainties in complex aerospace engineering systems. J. Sound Vib..

[296] Jung K.-H., Kim D.-H., Kim H.-J., Park S.-H., Jhang K.-Y., Kim H.-S. (2017). Finite element analysis of a low-velocity impact test for glass fiber-reinforced polypropylene composites considering mixed-mode interlaminar fracture toughness. Compos. Struct..

[297] Richardson M.O.W., Wisheart M.J. (1996). Review of low-velocity impact properties of composite materials. Compos. Part A Appl. Sci. Manuf..

[298] Evci C., Gülgeç M. (2012). An experimental investigation on the impact response of composite materials. Int. J. Impact Eng..

[299] Chiacchiarelli L.M., Cerrutti P., Flores-Johnson E.A. (2020). Compressive behavior of rigid polyurethane foams nanostructured with bacterial nanocellulose at low and intermediate strain rates. J. Appl. Polym. Sci..

[300] Simon U., Segelflugzeugbau A.S. (2019). Gliding Farther and Faster. ANSYS Advant. Mag..

[301] Waterman P. (2015). Composite Simulation Tips for New Users. Digit. Eng..

[302] Heimbs S., Strobl F. Crash Simulation of an F1 Racing Car Front Impact Structure. Proceedings of the 7th European LS-DYNA Conference.

[303] He X.D., Hong Y., Wang R.G. (2012). Hydroelastic optimisation of a composite marine propeller in a non-uniform wake. Ocean Eng..

[304] Ma S., Mahfuz H. (2012). Finite element simulation of composite ship structures with fluid structure interaction. Ocean Eng..

[305] Pemberton R., Summerscales J., Graham-Jones J. (2019). Marine Composites.

[306] Chen F., Liu L., Lan X., Li Q., Leng J., Liu Y. (2017). The study on the morphing composite propeller for marine vehicle. Part I: Design and numerical analysis. Compos. Struct..

[307] MESH Engineering & Software Co Resolving Vibration Induced Engineering Problems. http://www.mesh.com.tr/vibration-analyses.html..

[308] Forrister T. Analyzing Wind Turbine Blades with the Composite Materials Module. https://www.comsol.com/blogs/analyzing-wind-turbine-blades-with-the-composite-materials-module/.

[309] Weifei H. (2015). Reliability-Based Design Optimization of Composite Wind Turbine Blades for Fatigue Life under Wind Load Uncertainty.

[310] Barnes R.H., Morozov E.V. (2016). Structural optimisation of composite wind turbine blade structures with variations of internal geometry configuration. Compos. Struct..

[311] Oliveira M.N. (2019). De Powering Up for the Future. ANSYS Advant. Mag..

[312] (2013). Oil and Gas. ANSYS Advant. Mag..

[313] (2015). Oil and Gas. ANSYS Advant. Mag..

[314] Jha V., Latto J., Dodds N., Anderson T.A., Finch D., Vermilyea M. Qualification of Flexible Fiber-Reinforced Pipe for 10,000-Foot Water Depths. Proceedings of the Offshore Technology Conference.

[315] Duncan M., Niu H., Conley J.R., Edmondson S.J., Holub J., Andrenacci A., Gujare N. (2017). Flexible Reinforced Pipe and Reinforcement Tape. U.S. Patent.

[316] Chi P.D., Narayanaswamy S., Edmans B. Multiscale Analysis of Multilayer Composite Pipes. Proceedings of the 7th International Conference on Multiscale Materials Modeling.

[317] Guz I.A., Menshykova M., Paik J.K. (2017). Thick-walled composite tubes for offshore applications: An example of stress and failure analysis for filament-wound multi-layered pipes. Ships Offshore Struct..

[318] Seker B.S., Cakir F., Dogangun A., Uysal H. (2014). Investigation of the structural performance of a masonry domed mosque by experimental tests and numerical analysis. Earthq. Struct..

[319] Altunışık A.C., Genç A.F. (2017). Earthquake response of heavily damaged historical masonry mosques after restoration. Nat. Hazards Earth Syst. Sci..

[320] Varkonyi B., Belnoue J.P.-H., Kratz J., Hallett S.R. (2019). Predicting consolidation-induced wrinkles and their effects on composites structural performance. Int. J. Mater. Form..

[321] Najafgholipour M.A., Dehghan S.M., Dooshabi A., Niroomandi A. (2017). Finite Element Analysis of Reinforced Concrete Beam-Column Connections with Governing Joint Shear Failure Mode. Lat. Am. J. Solids Struct..

[322] Yang Z., Liu A., Yang J., Fu J., Yang B. (2020). Dynamic buckling of functionally graded graphene nanoplatelets reinforced composite shallow arches under a step central point load. J. Sound Vib..

[323] Khelifa M., Auchet S., Méausoone P.J., Celzard A. (2015). Finite element analysis of flexural strengthening of timber beams with Carbon Fibre-Reinforced Polymers. Eng. Struct..

[324] Scholz M.-S., Blanchfield J.P., Bloom L.D., Coburn B.H., Elkington M., Fuller J.D., Gilbert M.E., Muflahi S.A., Pernice M.F., Rae S.I. (2011). The use of composite materials in modern orthopaedic medicine and prosthetic devices: A review. Compos. Sci. Technol..

[325] Zou D., He T., Dailey M., Smith K.E., Silva M.J., Sinacore D.R., Mueller M.J., Hastings M.K. (2014). Experimental and computational analysis of composite ankle-foot orthosis. J. Rehabil. Res. Dev..

[326] Upender V., Srikanth E., Karthik G., Kumar N. (2018). Design Modeling and Optimization of a “Prosthetic Runner Blade. Natl. Tech. Res. Organ. India.

[327] Hamzah M., Gatta A. (2018). Design of a Novel Carbon-Fiber Ankle-Foot Prosthetic using Finite Element Modeling. IOP Conf. Ser. Mater. Sci. Eng..

[328] Jia Y., Wei X., Pu J., Xie P., Wen T., Wang C., Lian P., Xue S., Shi Y. (2019). A Numerical Feasibility Study of Kinetic Energy Harvesting from Lower Limb Prosthetics. Energies.

[329] Cahyono S.I., Anwar M., Diharjo K., Triyono T., Hapid A., Kaleg S. (2017). Finite element analysis of electric bicycle frame geometries. AIP Conf. Proc..

[330] Chidambaram P.K., Ramakrishanan R. (2014). Manufacturing, Testing of Polymer Nanocomposite and Analysis of Tennis Racket Frame. Int. J. Eng. Technol. Innov..

[331] Tryggvason H., Starker F., Lecomte C., Jonsdottir F. (2020). Use of dynamic FEA for design modification and energy analysis of a variable stiffness prosthetic foot. Appl. Sci..

[332] Tsao C.C., Hocheng H. (2012). Drilling processes for composites. Machining Technology for Composite Materials.

[333] Usui S., Wadell J., Marusich T. (2014). Finite Element Modeling of Carbon Fiber Composite Orthogonal Cutting and Drilling. Procedia CIRP.

[334] Yan X., Reiner J., Bacca M., Altintas Y., Vaziri R. (2019). A study of energy dissipating mechanisms in orthogonal cutting of UD-CFRP composites. Compos. Struct..

[335] Patel D.A., Buch V.R. (2018). Drilling of glass fiber reinforced polymer composite (GFRP): A review. Int. J. Recent Sci. Res..

[336] Fernández-Pérez J., Cantero J.L., Díaz-Álvarez J., Miguélez M.H. (2017). Influence of cutting parameters on tool wear and hole quality in composite aerospace components drilling. Compos. Struct..

[337] Rahme P., Landon Y., Lachaud F., Piquet R., Lagarrigue P. (2015). Delamination-free drilling of thick composite materials. Compos. Part A Appl. Sci. Manuf..

[338] Nikishkov Y., Makeev A., Seon G. (2013). Progressive fatigue damage simulation method for composites. Int. J. Fatigue.

[339] ANSYS Inc Composite Materials for Aerospace. https://www.ansys.com/solutions/solutions-by-industry/high-tech/composite-materials-for-high-performance-electronics.

[340] Gerofi B., Ishikawa Y., Riesen R., Wisniewski R.W. (2019). Operating Systems for Supercomputers and High Performance Computing.

[341] Zhai S., Zhang P., Xian Y., Zeng J., Shi B. (2018). Effective thermal conductivity of polymer composites: Theoretical models and simulation models. Int. J. Heat Mass Transf..

[342] Ngo I.-L., Byon C. (2015). A generalized correlation for predicting the thermal conductivity of composites with heterogeneous nanofillers. Int. J. Heat Mass Transf..

[343] Ngo I.L., Byon C., Lee B.J. (2018). Analytical study on thermal conductivity enhancement of hybrid-filler polymer composites under high thermal contact resistance. Int. J. Heat Mass Transf..

